# A multi-scale eco-evolutionary model of cooperation reveals how microbial adaptation influences soil decomposition

**DOI:** 10.1038/s42003-020-01198-4

**Published:** 2020-09-21

**Authors:** Elsa Abs, Hélène Leman, Régis Ferrière

**Affiliations:** 1 Department of Ecology and Evolutionary Biology, University of California, Irvine, CA 92697 USA; 2 Interdisciplinary Center for Interdisciplinary Global Environmental Studies (iGLOBES), CNRS, Ecole Normale Supérieure, Paris Sciences & Lettres University, University of Arizona, Tucson, AZ 85721 USA; 3 Numed Inria team, UMPA UMR 5669, Ecole Normale Supérieure, Lyon, 69364 France; 4 Centro de Investigación en Matemáticas, Guanajuato, 36240 Mexico; 5 Department of Ecology and Evolutionary Biology, University of Arizona, Tucson, AZ 85721 USA; 6 Institut de Biologie (IBENS), Ecole Normale Supérieure, Paris Sciences & Lettres University, CNRS, INSERM, Paris, 75005 France

**Keywords:** Microbial ecology, Evolutionary theory

## Abstract

The decomposition of soil organic matter (SOM) is a critical process in global terrestrial ecosystems. SOM decomposition is driven by micro-organisms that cooperate by secreting costly extracellular (exo-)enzymes. This raises a fundamental puzzle: the stability of microbial decomposition in spite of its evolutionary vulnerability to “cheaters”—mutant strains that reap the benefits of cooperation while paying a lower cost. Resolving this puzzle requires a multi-scale eco-evolutionary model that captures the spatio-temporal dynamics of molecule-molecule, molecule-cell, and cell-cell interactions. The analysis of such a model reveals local extinctions, microbial dispersal, and limited soil diffusivity as key factors of the evolutionary stability of microbial decomposition. At the scale of whole-ecosystem function, soil diffusivity influences the evolution of exo-enzyme production, which feeds back to the average SOM decomposition rate and stock. Microbial adaptive evolution may thus be an important factor in the response of soil carbon fluxes to global environmental change.

## Abstract (web summary)

Abs et al. develop a multi-scale model to explain the evolution of microbial cooperation driving the decomposition of soil organic matter. Their model shows that the evolutionary stability of decomposition depends on a combination of local extinctions, microbial dispersal, and limited soil diffusivity.

## Introduction

Microorganisms drive critical ecosystem processes, such as nutrient mineralization and the decomposition of organic matter^1^. Many of these processes depend on the conversion of complex compounds into smaller products that microbes can assimilate for growth and maintenance. Except in environments where simple nutrients are abundant, microbes rely on extracellular enzymes (exoenzymes) to perform this conversion^2^. By doing so, they face a “public good spatial dilemma”^3,4^. The “spatial dilemma” arises because the public good involves costly compounds (exoenzymes) that are secreted outside the cell; the products of decomposition may diffuse away from the enzyme-secreting microbe and therefore benefit not only the individuals producing exoenzymes, but also neighboring cells^5,6^. Evolutionary theory predicts that producers of public goods are vulnerable to cheating by individuals that receive the benefits without paying the cost of production. Without some mechanism to stabilize cooperation against cheating^7^, the evolutionary loss of public goods production is expected. The problem is made even worse by diffusion^3,4^. Nonetheless, the microbial world provides ubiquitous examples of diffusive public goods, e.g. siderophores that scavenge iron^8–11^, polymers that enable biofilm formation^12^, and allelopathic compounds that reduce competition^13^. Conditions must exist that promote the evolution of exoenzyme production in spite of diffusion.

Evolutionary game theory provides a powerful framework for investigating conditions that favor exoenzyme production^14–16^. Evolutionary game-theoretic models have been developed to address competition between exoenzyme-producing and non-producing strains^17–20^. Considering the diffusivity of products, these models have highlighted the importance of ecosystem spatial heterogeneity for the evolution of the production mechanism. For example, organic substrates, microbes, and mineral particles form a three-dimensional matrix of aggregates and pore spaces of different sizes in soils^21^. For enzyme-dependent microbes, the soil structure should influence the movement of substrates, enzymes, and usable products^22^, and, as a consequence, the fate of cheating microbes^17,23,18^.

Our understanding of the evolutionary stability of diffusive public goods in general, and of degradative enzyme production in particular, remains incomplete. One limitation of previous models is their focus on two-way competition between two strains, typically a producing strain and a non-producing or “pure cheater” strain. A key issue here is that mechanisms that promote stability of producers against pure cheaters might fail to prevent “erosion” of cooperation by mutant strains that produce slightly less of the public good than the wild-type, or resident strain^24^. Pure cheaters may go locally extinct when they do not receive enough resource produced by cooperators; however, strains that produce less, rather than none, of the public good should be less sensitive to the harm they inflict to the community^25^. On the other hand, producers may be vulnerable to pure cheaters and yet resist invasion by strains that invest only slightly less into the common good. We thus expect conditions for the evolutionary stability of cooperation to be different when considering the recurrent events of mutation of small effects and selection that shape the evolutionary trajectory of exoenzyme production.

To predict the outcome of selection on small-effect variants, we need to evaluate the population growth rate of initially rare mutant types interacting with any given resident type. To achieve this, previous models of microbe-enzyme systems need to be revisited and extended, so that invasion fitness of small-effect mutants can be computed^26,27^. To describe the interaction of resident strains with mutant cells, which, initially at least, occur in small, spatially localized populations, individual-level modeling of microbe-enzyme systems is required. Previous microbe-enzyme ecological models (reviewed in refs. ^28,29^) are phenomenological, rather than derived by scaling up from microscopic processes acting locally at the level of individual entities. The main difficulty here is to address the extremely different scales that characterize the entities (cells, enzymes, substrates, products) and processes that entangle them. Here we derive a hybrid, stochastic-deterministic model that takes this multiplicity of scales into account. By applying the hybrid model to a spatially structured habitat, we elucidate conditions that promote the evolutionary convergence and stability of exoenzyme production. We show that resource diffusivity is a strong control of the selection gradient of exoenzyme production, which feeds back ecologically to determine the average soil decomposition rate and carbon stocks of the whole system. These results suggest that the adaptive evolution of microbial exoenzyme production may be an important factor in the response of soil decomposition to environmental changes that affect soil properties.

## Results

### Model overview

To construct a spatially explicit model of microbe-enzyme decomposition, we focus on cells and unprotected soil organic carbon (SOC)^30^ and we assume nitrogen and phosphorus to be non-limiting. Space is modeled as a two-dimensional lattice of microsites, with each microsite potentially occupied by a population of cells. Decomposition is seen as a microbial public good game, whereby individual microorganisms invest resources into the production of degradative exoenzymes. Exoenzyme molecules bind SOC molecules and catalyze the depolymerization of SOC into dissolved organic carbon (DOC) molecules. DOC molecules occurring in a microsite may be uptaken and metabolized by cells present in the microsite, resulting in cell growth and exoenzyme production. The fraction of uptaken DOC that is invested by a cell into exoenzyme production, as opposed to cell biomass production, is denoted by *φ*. This is the focal trait that characterizes the microbial phenotype, for which we assume heritable variation, originating in mutation^31,32^.

### Ecosystem dynamics at microsite scale

We assume that cells, enzymes, substrates (SOC), and products (DOC) are well mixed within each microsite. Processes operating at the level of individual entities are cell division, determined by accrued and stored resources reaching a threshold within the cell; cell death and enzyme degradation, at constant rates; cell carbon consumption to cover the structural and energetic costs of producing cell tissues and enzyme molecules; SOC–enzyme complex formation followed either by dissociation into one SOC and one enzyme, or by depolymerization yielding the equivalent number of DOC molecules and releasing the enzyme (i.e. successful decomposition). Additional processes operating at the level of microsites are external inputs of SOC and DOC, losses of SOC and DOC (leaching), diffusion of DOC, and random extinction of the local population. The extinction of a local population is modeled as the simultaneous death of all the individuals within the microsite; the dead organic matter is then partly leached and partly recycled into SOC and DOC, as it is for individual cell death. Local extinction events account for environmental stochasticity at microsite scale, which is known to contribute to the evolutionary stability of dispersal^33–36^.

**Fig. 1:**
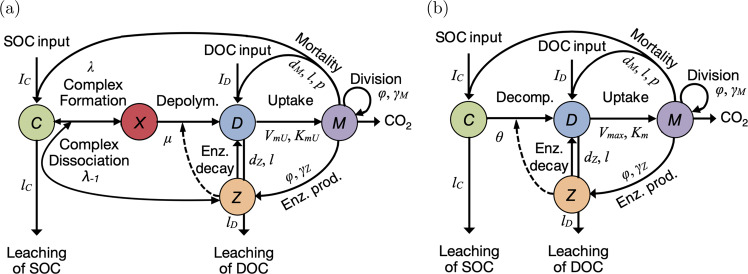
Microbe-enzyme-driven decomposition of soil organic matter: agents and processes. **a** Five-compartment model, with soil organic carbon (SOC, *C*), dissolved organic carbon (DOC, *D*), microbial cells (*M*), enzymes (*Z*), and SOC–enzyme complexes (*X*). (b), Four-compartment model, without the SOC–enzyme complexes (*X*). Plain arrows indicate carbon fluxes among compartments and in and out of the system. Dotted arrows indicate the exoenzyme concentration dependence of the decomposition rate.

**Fig. 2:**
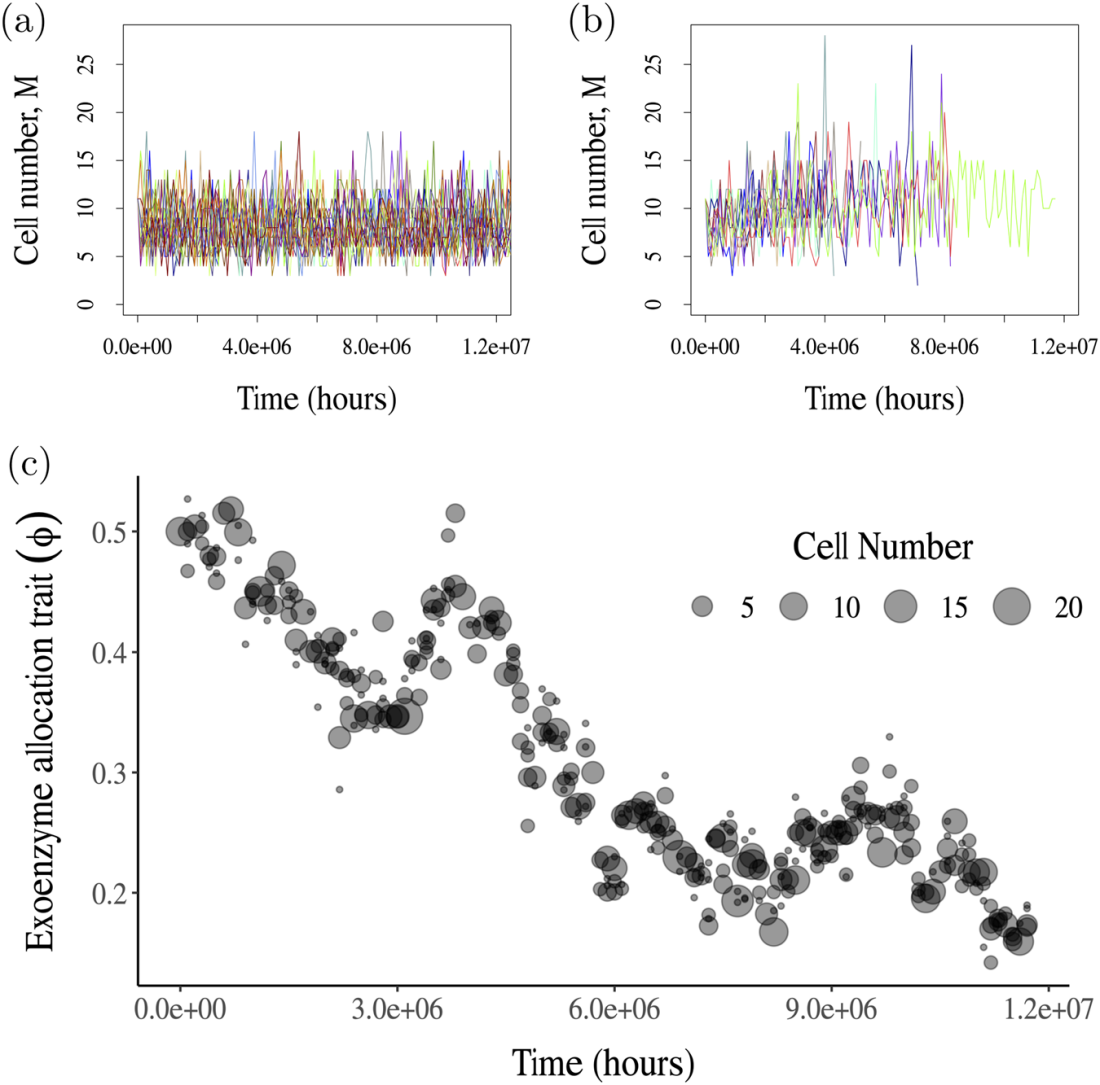
Dynamics of the cell population size and microbial trait *φ*, with and without mutation. **a** Cell population dynamics without mutation. Due to demographic stochasticity, the populations fluctuate around the deterministically predicted steady state of 10 individuals. **b** Cell population dynamics with mutation, with probability *p*_mut_ = 0.1. As populations evolve, they reach the minimum viable value of the enzyme production trait, *φ*, and go extinct in most of the 10 runs. **c** Evolution of enzyme allocation fraction, *φ* in one of the simulations from (**b**). In (**a**) and (**b**), 10 simulation runs are shown. All simulations are for a finite microbial population in a single microsite. In all simulations, initial cell population was monomorphic with *φ* = 0.5, and the four variables *c*, *d*, *M*, *z* were initialized at the steady state values predicted by the deterministic model with *φ* = 0.5. All constant parameters are set to the default values (Table 1), except ${T}_{\max }=1{0}^{8}$.

**Fig. 3:**
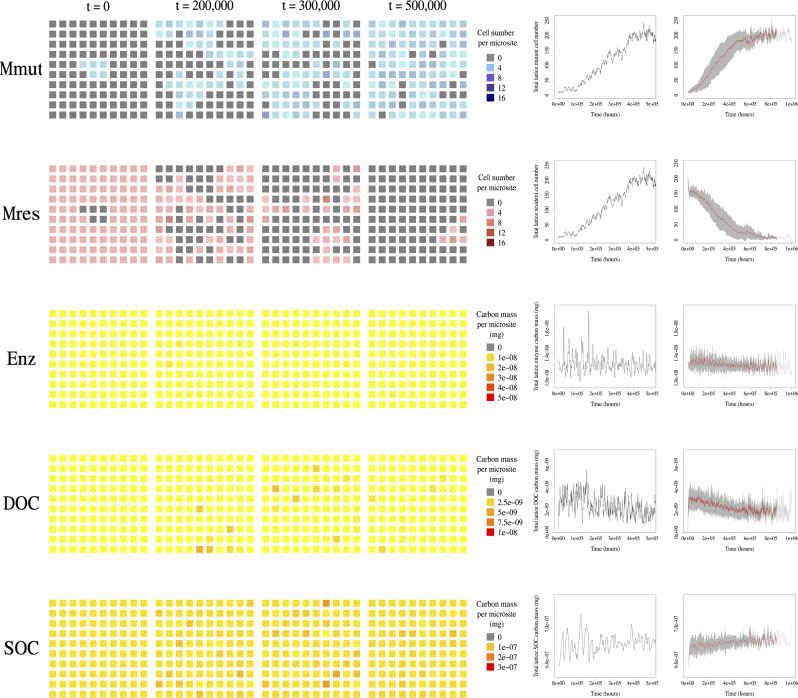
Spatio-temporal dynamics of invasion of a mutant cooperator (*φ*_mut_ = 0.8) into the ecosystem established by a resident strain investing slightly less in exoenzyme production (*φ*_res_ = 0.75). From top to bottom: temporal dynamics of the mutant cell population (*M*_mut_), resident cell population (*M*_res_), enzyme (*Enz*), DOC (*DOC*), SOC (*SOC*). Units are number of cells for *M*_mut_ and *M*_res_, and mg for enzyme, DOC and SOC. Columns 1–4: example simulation run of the spatial hybrid stochastic-deterministic model over a 10 × 10 lattice of microsites, snapshots from time *t* = 0 to *t* = 5 × 10^5^. Color-coded scales were chosen to span the whole range of the corresponding variables over the whole simulation. Columns 5 and 6 show the total number of cells and total mass over time. Column 5: Same simulation run as Columns 1–4. Column 6: Mean trajectories averaged over 20 replicated simulation runs (red line), and variance (gray area). All constant parameters are set to the default values (Table 1). The lattice was initialized with all microsites occupied by residents, except for five microsites occupied by mutants at the center of the lattice. All ecosystem variables *z*, *c*, *d*, and *M* were fixed at the steady state determined by the established resident strain. See “Methods” (subsection “Simulation algorithm for the hybrid spatial model”) for simulation detail.

**Fig. 4:**
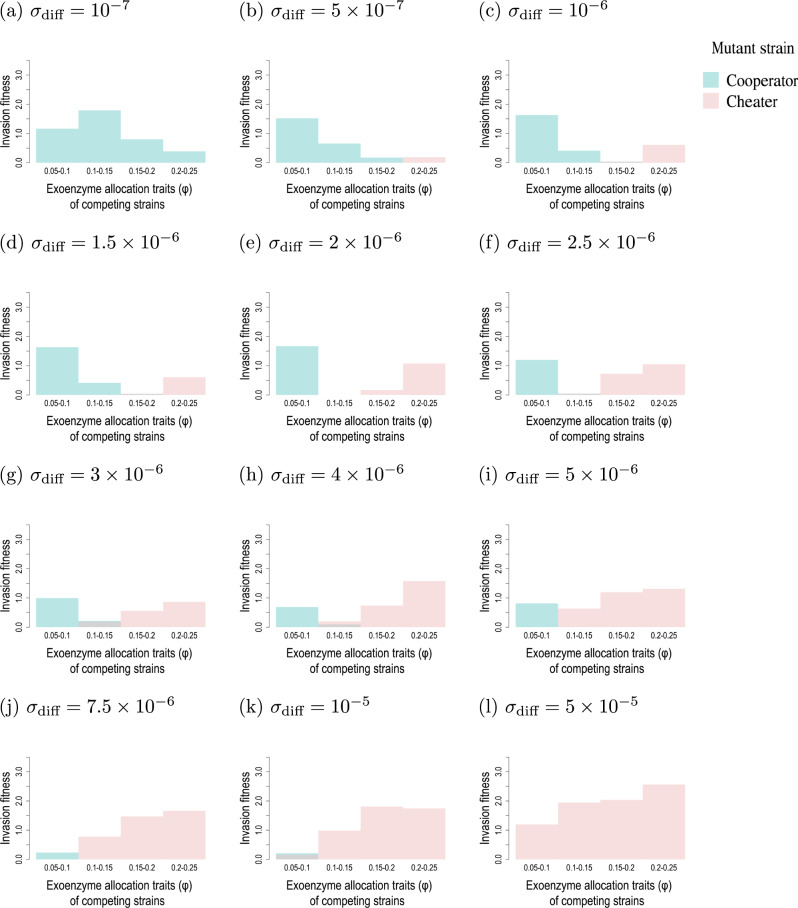
Patterns of selection on exoenzyme production at different soil diffusion rates. Each graph shows the mutant invasion fitness across pairwise resident-mutant competing strains. Invasion fitness is measured as the product of the mutant survival probability and the average long-term growth rate of growing mutant populations among stochastic simulation replicates, rescaled by multiplying by ${T}_{\max }$. The survival probability is estimated as the fraction of simulations with a non-extinct mutant population at ${T}_{\max }$. The long-term growth is calculated as the average of $(1/{T}_{\max})\mathrm{log}\,\frac{{\rm{final}}\,{\rm{mutant}}\,{\rm{population}}\,{\rm{size}}}{{\rm{initial}}\,{\rm{mutant}}\,{\rm{population}}\,{\rm{size}}}$ among the 20 runs for each pairwise competition test, with ${T}_{\max }=1{0}^{6}$.  Pink bars show invasion fitness of the cheater strain taken as mutant (with the lower *φ* value in the competing pair); blue bars show invasion fitness of the cooperator strain taken as mutant (with the higher *φ* value in the competing pair). Positive invasion fitness of cheater mutants (pink bars) indicate selection against exoenzyme production. Positive invasion fitness of cooperator mutants (blue bars) indicate selection in favor of exoenzyme production. For each diffusion rate, the ESS is bracketed between the maximum *φ* value for which the cooperator mutant has positive invasion fitness, and the minimum *φ* value for which the cheater mutant has positive invasion fitness. All constant parameters are set to the default values (Table 1). Mutant initial population size is set to 5% of the abundance of the resident population in the central microsites. We tested values of *σ*_d*iff*_ between 10^−8^ and 10^−4^ and report results for *σ*_d*iff*_ between 10^−7^ and 5 × 10^−5^ as variation of *σ*_d*iff*_ outside this range had no effect.

**Fig. 5:**
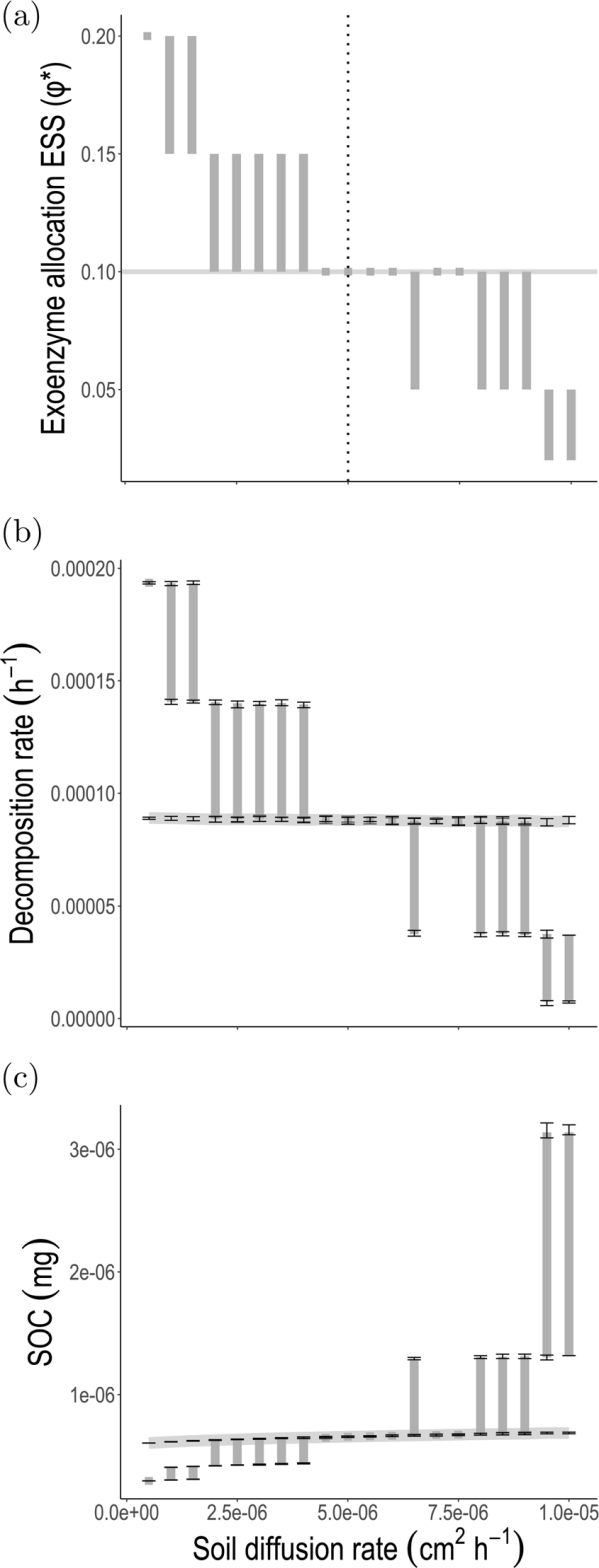
Effect of soil diffusion on the evolution of exoenzyme production and lattice-scale feedback on ecosystem function (decomposition rate and soil carbon stock), predicted by the spatial hybrid model. **a** Bars, Enxoenzyme allocation ESS as a function of diffusion. Continuous line, Exoenzyme allocation fixed at ESS predicted for intermediate diffusion rate (*σ*_diff_ = 5 × 10^−6^), with no adaptation to the other diffusion rate values. **b** Bars, Feedback of exoenzyme allocation adaptation to lattice-scale decomposition. Continous line, Lattice-scale decomposition as a function of diffusion, without microbial evolutionary adaptation. **c** Bars, Feedback of exoenzyme allocation adaptation to lattice-scale carbon stock. Continuous line, Lattice-scale carbon stock as a function of diffusion, without microbial evolutionary adaptation. In (**a**), for each diffusion rate the ESS estimation is bounded between the maximum *φ* value for which the cooperator mutant has positive invasion fitness, and the minimum *φ* value for which the cheater mutant has positive invasion fitness (see Fig. 4). In (**b**) and (**c**), for each diffusion rate the exoenzyme allocation fraction is fixed at its corresponding ESS from (a). We then ran simulations of a monomorphic microbial population at ESS and calculated the decomposition rate (total enzyme mass multiplied by parameter *θ*, which is proportional to the decomposition rate, see its exact biological interpretation in Table 1) and the SOC mass per microsite averaged across the lattice and over time (between time 2 × 10^5^ and ${T}_{\max }=1{0}^{6}$, to remove the initial transient). Error bars measure variation of the minima and maxima among simulations due to process stochasticity. All constant parameters are set to the default values (Table 1). See “Methods” (subsection “Simulation algorithm for the hybrid spatial model”) for simulation detail.

We measure the abundance of all entities in units of carbon mass. The local dynamics of decomposition within a microsite involves fluxes in and out of five local compartments: SOC (*C*), DOC (*D*), cells (biomass *M*), enzymes (*Z*), and SOC–enzyme complexes (*X*) (Fig. 1a). To scale up the dynamics of decomposition from microscopic, stochastic processes, we take the following steps:

- Step 1: We define the stochastic processes acting at the level of *C*, *D*, *M*, *Z*, *X* entities (molecules, cells) that make the “CDMZX” model (Fig. 1a and subsection “Construction of the five-compartment model” of “Methods”).
- Step 2: We apply appropriate rescaling on the rates of complex (*X*) formation, dissociation or decomposition, to express that complex dissociation and complex decomposition are much faster than complex formation. By doing so, we reduce the stochastic model to four-state variables (*C*, *D*, *M*, *Z*) that make the “CDMZ” model (Fig. 1b and Supplementary Information 3.1).
- Step 3: We rescale the reduced stochastic model into a stochastic-deterministic model, in which only *M* is treated as an integer-valued variable. This is consistent with the assumption that a cell is of the order of 10^7^ times larger in units of carbon mass than one enzyme or substrate (SOC) molecule, and 10^10^ times larger than one product (DOC) molecule; and that in a given volume, the number of cells is between 10^−5^ and 10^−10^ times smaller than the number of molecules of SOC, DOC or enzyme. As a consequence, the events affecting *C*, *D*, and *Z* are much faster and more frequent than events affecting *M*, allowing us to treat the dynamics of *C*, *D,* and *Z* as deterministic over time bouts of constant cell population size. We denote deterministic state variables with lower case, *c*, *d,* and *z*. Mathematically, the resulting stochastic-deterministic model is a Piecewise Deterministic Markov Process, or “PDMP” (Supplementary Information 3.2).
- Step 4: Finally, we simplify the PDMP model further by noting that the growth of individual cells is driven by events (resource uptake) that occur on the same timescale as the events affecting SOC, DOC, and enzymes in the stochastic CDMZ model defined at step 2. Then the consumption of DOC by cells is no longer a stochastic process but instead depends on *M* deterministically. Cell production thus becomes nearly deterministic, and the only remaining stochastic process is cell death (which is why we retain the *M* notation for cells). Even though the rigorous proof of step 4 is beyond the scope of the paper, we will adopt the approximation—that we call the “hybrid” model—as we develop the spatially explicit extension of the model. Note that all the simulations presented in this article (Figs. 2–5) are based on the hybrid model and its spatial extension described in the next subsection.

In the hybrid model, the ecosystem dynamics are driven by jumps of the finite number of cells *M* (corresponding to the cell death events), interspersed with periods of continuous cell growth and change in the abundance of SOC, DOC, and enzyme. Cell death occurs at random times, at rate *d*_*M*_. When a cell dies, it is removed from the system and its carbon content is recycled into SOC and DOC. Birth events occur deterministically once the cell has experienced enough resource uptake events to assimilate and store the amount of DOC corresponding to one cell. Step 4 allows us to model the amount *S*_*i*_ of biomass stored within cell *i* as governed by

$$\frac{{\rm{d}}{S}_{i}(t)}{{\rm{d}}t}=(1-\varphi ){\gamma }_{M}{V}_{\max }\frac{d}{{K}_{m}+d}{\omega }_{M},$$

where *d* measures the rescaled dynamics of DOC, in carbon mass unit, and *ω*_*M*_ is the carbon mass content of a cell that does not store additional carbon for upcoming division. Once *S*_*i*_ reaches *ω*_*M*_, the cell divides and both mother and daugther cells’ reserve is set back to 0. The other parameters are *φ* (see Supplementary Fig. 2 for the effect of *φ* on dynamics of *c*, *d*, *M*, *z*), the fraction of investment in exoenzyme production vs. cell growth; *γ*_*M*_, the microbial carbon mass production fraction, or microbial growth efficiency (MGE); ${V}_{\max }$ and *K*_*m*_, the maximum uptake rate and uptake half-saturation constant, respectively, of the Michaelis–Menten uptake function.

For a given number of cells, *M*, the change in enzyme, SOC and DOC are governed by

$$\left\{\begin{array}{l}\,\dot{c}(t)={I}_{C}-{l}_{C}c-\theta zc,\hfill\\ \dot{d}(t)={I}_{D}-{l}_{D}d+\theta zc+(1-l){d}_{Z}z-{V}_{\max }\frac{d}{{K}_{m}{\,}+{\,}d}{\omega }_{M}M,\\ \dot{z}(t)=\varphi {\gamma }_{Z}{V}_{\max }\frac{d}{{K}_{m}{\,}+{\,}d}{\omega }_{M}M-{d}_{Z}z,\hfill\end{array}\right.$$

where *c*, *d*, *z* are in units of carbon mass, *I*_*C*_ and *I*_*D*_ are the external inputs of SOC and DOC, respectively, from litter, *l*_*C*_ and *l*_*D*_ are the leaching rates of SOC and DOC to deeper soil layers, *θ*, which is proportional both to the encounter rate of enzymes and SOC and to the decomposition rate (see its exact biological interpretation in Table 1), *l* is the fraction of deactivated *z* that is leached instead of recycled, *γ*_*Z*_ is the enzyme carbon mass production fraction, and *d*_*Z*_ is the rate at which one enzyme carbon gram permanently loses its capacity to bind to SOC (deactivation rate). We assume litter inputs and leaching to be homogeneous at the spatial scale considered here (cm^3^). The different notations, uppercase for cells and lower case for SOC, DOC, and enzymes, are meant to indicate that cell abundance is stochastic and in number of individuals, while the latter measure the rescaled continuously-varying amounts in carbon mass unit.

**Table 1:** Parameters of the deterministic model in biomass.

Parameter	Unit	Description	Default value
*V*	cm^3^	Unit soil volume of one cell	10^−9^
*k*		System size	10
*k* × *V*	cm^3^	Volume of one microsite	10^−8^
*L* × *L*		Lattice size in number of microsites	10 × 10
*φ*		Enzyme allocation fraction	[0, 1]
*γ*_*M*_		Microbial carbon biomass production fraction	0.3
*γ*_*Z*_		Enzyme carbon mass production fraction	0.4
*ω*_*M*_	mg	Mass of 1 cell	10^−9^
*ω*_*Z*_	mg	Mass of 1 enzyme molecule	10^−16^
*ω*_*C*_	mg	Mass of 1 SOC molecule	10^−16^
*ω*_*D*_	mg	Mass of 1 DOC molecule	10^−19^
*α*		Structural cost in DOC of 1 cell	10^10^
$\alpha ^{\prime}$		Energetic cost in DOC of 1 cell	2.33 × 10^10^
*β*		Structural cost in DOC of 1 SOC molecule	10^3^
*ρ*		Structural cost in DOC of 1 enzyme molecule	10^3^
$\rho ^{\prime}$		Energetic cost in DOC of 1 enzyme molecule	1.5 × 10^3^
*d*_*M*_	h^−1^	Microbial carbon biomass death rate	2 × 10^−4^
*d*_*Z*_	h^−1^	Enzyme carbon mass deactivation rate	2 × 10^−3^
${V}_{\max }$	h^−1^	Maximum uptake rate (in carbon mass)	0.42
*θ*	mg^−1^ h^−1^	(Encounter probability of two given SOC and enzyme molecules) × (decomposition rate)	7 × 10^5^
*K*_*m*_	mg	Uptake half-saturation constant	3 × 10^−10^
*I*_*C*_	mg h^−1^	External input of SOC	5 × 10^−13^
*I*_*D*_	mg h^−1^	External input of DOC	0
*l*_*C*_	h^−1^	SOC leaching rate	10^−6^
*l*_*D*_	h^−1^	DOC leaching rate	10^−6^
*l*		Fraction of dead cell and deactivated enzyme leached instead of recycled	0
*p*		Fraction of recycled dead cell flowing into SOC (remaining fraction flows into DOC)	0.5
${T}_{\max }$	h	Maximum simulation time	10^6^
*p*_*disp*_		Probability of dispersal of a new cell	0.3
*p*_*open*_		Probability of local extinction due to a micro-disturbance	0.01
*p*_*mut*_		Probability of mutation per cell division event	between 1/(*K*ln(*K*)) and 1/K^2^
*σ*_*mut*_		Standard deviation of mutation effect	[0.01−0.1]
*σ*_*diff*_		DOC diffusion rate between 2 microsites	[1 × 10^−7^–5 × 10^−5^]

### Spatial extension of ecosystem dynamics to lattice scale

In order to model the process of mutant invasion in a resident population of cells, we extend the hybrid model to a spatially explicit, spatially homogeneous square regular lattice of microsites. Spatial homogeneity means that all microsites have the same volume and the same abiotic parameters, *I*_*C*_, *I*_*D*_, *l*_*C*_, *l*_*D*_, and *l*. We couple hybrid models among microsites by accounting for the diffusion of products (DOC) and dispersal of cells between adjacent microsites. The DOC diffusion between microsites is modeled by approximating a continuous diffusion with a Euler scheme in which time is discretized with a fixed time step interval, *τ*_diff_. At each time, a step of the Euler scheme associated with the diffusion equation

$$\frac{{\rm{d}}}{{\rm{d}}t}d({\bf{x}},t)={\sigma }_{{\rm{diff}}}\Delta d({\bf{x}},t)$$

is realized for the variable *d*, where **x** is the spatial position and *σ*_diff_ is the DOC diffusion coefficient. Space discretization in the Euler scheme is chosen to match the habitat lattice structure. Cell dispersal may occur following birth events. The daughter cell disperses with probability *p*_disp_ to one of the four neighboring microsites, or it is added to the mother cell population with probability 1 − *p*_disp_. If one or more of the adjacent microsites is empty, the dispersing cell moves to one of them with equal probability. If all neighboring microsites are occupied, there is a probability *p*_open_ that a micro-disturbance of the soil strikes and opens one of the microsite by killing the local cell population therein, which then becomes occupied by the dispersing cell; the corresponding SOC and DOC released by the dead cells are recycled locally. If no microsite opens (with probability 1−*p*_open_), the dispersal event is unsuccessful and the daughter cell remains in its maternal microsite. The dynamics of each microsite is recalculated between two diffusion steps and after each cell birth or death event. See “Methods” (subsection “Simulation algorithm for the hybrid spatial model”) for further detail.

### Eco-evolutionary dynamics

Adaptive evolution of the exoenzyme allocation fraction trait, *φ*, is modeled by considering trait mutations that cause the trait of daughter cells to differ from the maternal trait value. The value of a mutated trait is assumed to be normally distributed around the maternal value, with small variance to represent mutations of small effect. Cells that have the same *φ* value belong to the same “strain”; relative to a given strain, we call “cheaters” mutants that invest less in exoenzyme production (smaller *φ*) and “cooperators” mutants that invest more in exoenzyme production (larger *φ*). Any new mutant strain arises in an ecological environment (characterized by the abundance of SOC, DOC, and exoenzymes) that has been shaped by all other co-occurring strains. Selection occurs because strains with different *φ* will differentially succeed at acquiring the DOC resource for which they compete. The direction and strength of selection on the evolving trait is then predicted by invasion fitness (intrinsic population growth rate from low density) of a mutant strain competing against a resident strain^27,26^.

Based on the mathematical theory of adaptive dynamics^37,38^, the system’s eco-evolutionary dynamics may converge in the long run towards a stable distribution of trait values. If mutation effects are small enough, theory predicts conditions under which selection is stabilizing; when that is the case, the evolved trait distribution will typically have a narrow range, with the expected mean predicted as an attractive “evolutionarily stable strategy” (ESS). An ESS is a trait value that no nearby mutant can invade. When mutants closer to the ESS outcompete their resident progenitor, then the ESS is attractive and the system’s eco-evolutionary dynamics converge to the ESS. In this case, the (attractive) ESS predicts the outcome of the full eco-evolutionary dynamics under the assumption of mutations of small effects^37^.

First, we analyze the evolutionary model at the microsite scale, in which all entities are being well mixed and birth occurs with mutation. This analysis shows that cooperation (enzyme production) is always counter-selected in favor of cheaters (Fig. 2). Next we analyze the model at the lattice scale. To determine the direction of selection on enzyme allocation (Figs. 3,4), we run invasion assays of a given mutant introduced at low density in the lattice occupied by a stationary population of a resident strain (without mutation). Compared to the microsite scale, the lattice scale adds diffusion of DOC molecules, cell dispersal, and micro-disturbances that kill cell populations in randomly targeted microsites. From these invasion assays we can predict the outcome of enzyme allocation evolution at lattice scale. Finally, to determine the decomposition rate and equilibrium SOC stock corresponding to the adapted value of enzyme allocation (Fig. 5), we run the spatial model with the adapted strain and no mutation.

### Eco-evolutionary dynamics at single-site scale: extinction

In a single, isolated microsite occupied by a large population, ecosystem dynamics can be approximated by the deterministic “cdmz” model (equation S.5 in Supplementary Information 3.3), and the selection gradient of the exoenzyme allocation trait can easily be derived. At the (non-trivial) ecological equilibrium (*c*, *d*, *m*, *z*) of a resident population with trait value *φ*, the growth rate of a mutant strain with trait value $\varphi ^{\prime}$ is $(1-\varphi ^{\prime} ){\gamma }_{M}{V}_{\max }\frac{d}{{K}_{m}{\,}+{\,}d}-{d}_{M}$, hence the selection gradient of *φ* (derivative of the mutant long-term growth rate with respect to the mutant trait, evaluated at the resident trait value) is equal to $-{\gamma }_{M}{V}_{\max }\frac{d}{{K}_{m}{\,}+{\,}d}$. For any parameter combination and value of the DOC resident equilibrium *d*, this quantity is always negative: investing in exoenzyme production is always selected against. Thus, any initial level of microbial cooperation will be gradually eroded by the process of mutation-selection, driving the population towards the threshold trait value at which extinction occurs—an instance of evolutionary suicide^39–41^.

In finite populations, mutant success or failure becomes probabilistic. Due to random genetic drift, cheater phenotypes may fail to invade, and cooperator mutants may occasionally go to fixation. Long-term adaptive dynamics driven by rare mutation and selection in finite populations have been studied in a general framework by Champagnat et al.^42^. They showed that the evolutionary trait dynamics can be described mathematically as a diffusion process whereby a Brownian motion (white noise) is added to a trend driven by the deterministic selection gradient. To illustrate these general results, Fig. 2 shows simulations of a finite microbial population in a single microsite, without and with adaptive evolution of exoenzyme production. In the absence of evolution, simulated populations tend to persist over the total computation time. With evolution, simulated populations generally go extinct within that same time frame. In spite of fluctuations due to random genetic drift, adaptive evolution drives the exoenzyme production trait towards the threshold at which the microbial population becomes non-viable.

### Eco-evolutionary dynamics at lattice scale: stability

We address the evolution of exoenzyme production in spatially extended ecosystems using the spatial version of the hybrid model. On a lattice, exoenzyme producers may resist invasion by non-producer mutants because of the non-uniform distribution of cell types that emerges across microsites, due to local cell dispersal. We ran simulations of the spatial model to test the consequences of this mechanism for the evolution of exoenzyme production as a continuous trait, as opposed to an all-or-nothing character (as in previous studies).

Spatial segregation of resident and mutant strains across microsites is key to the evolutionary stability of exoenzyme production. If dispersal were to mix resident and mutant strains within microsites, any slightly cheating mutant would always invade and spread across the lattice. This is because the diffusion of DOC creates local conditions (within microsites) that are even more unfavorable to the resident strain than in the case of a single, isolated microsite. The long-term consequence is evolutionary suicide, as in the case of a well-mixed population. In contrast, with dispersal tied to micro-disturbances, resident and mutant strains do not mix within microsites. Thus, the local resource pool (DOC) to which cells of a given strain have access is entirely determined by their own exoenzyme production, and the diffusion of DOC from nearby microsites. The local growth of a strain then determines its chance of colonizing nearby empty microsites and spreading across the lattice.

Depending on the DOC diffusion rate, spatial segregation of strains at microscale can promote the persistence of exoenzyme-producing strains against invasion by cheater strains that produce slightly less (negative selection against cheating); and favor invasion by even stronger exoenzyme producers (positive selection for cooperation). Figure 3 shows an example of the latter. To further evaluate the effect of diffusion on the selection gradient of exoenzyme production, we measured the invasion fitness of mutant strains in pairwise competition with slightly different resident strains, across a range of soil diffusion rates, *σ*_diff_.

In Fig. 4, pairwise competition simulations run across the trait range 0.05–0.25, in increments of 0.05, and under different values of the soil diffusion rate, show a clear pattern of directional selection for increasing exoenzyme production when soil diffusion is low (cooperator mutants have positive fitness), and directional selection for decreasing *φ* towards zero when soil diffusion is high (cheater mutants have positive fitness). Supplementary Fig. 3 shows that we obtain the same trend with different lattice size.

ESS values can be approximated from the numerical results reported in Fig. 4. For a given value of the soil diffusion rate, the ESS is bracketed between the maximum *φ* value for which the cooperator mutant has positive invasion fitness, and the minimum *φ* value for which the cheater mutant has positive invasion fitness. For example, for *σ*_diff_ = 3 × 10^−6^, the ESS is bracketed in (0.1–0.15); for *σ*_diff_ = 7.5 × 10^−6^, the lower and upper values of the interval are equal to 0.1, which is the approximated value of the ESS. ESS approximated for different values of soil diffusion are shown in Fig. 5a. For high diffusion, exoenzyme production is counter-selected across the whole range of *φ* values. For intermediate to low diffusion, there exists a viable exoenzyme production ESS and starting from very low exoenzyme production, the system is predicted to evolve towards the ESS. The lower the soil diffusion rate, the larger the exoenzyme production ESS.

### Microbial adaptation and feedback to decomposition

In nature, parameters such as the diffusion rate may depend on environmental features such as soil properties and precipitation, that can vary widely across ecosystems. We find that diffusion has a strong influence on the selection gradient of exoenzyme production (Fig. 4). To further characterize this influence and investigate its ecosystem-level functional consequences, we extracted the pattern of variation of the exoenzyme production ESS for several values of diffusion rates (Fig. 5a) and computed the corresponding decomposition rate (Fig. 5b) and SOC equilibrium stock (Fig. 5c) per microsite averaged across the lattice. The different values of diffusion could represent spatial variation across ecosystems, or a temporal sequence driven by some external environmental factor, e.g. a gradual change in precipitation.

Figure 5 shows a clear departure of all three outputs from the non-adaptive scenario (continuous horizontal line) at low and high diffusion. Decreasing diffusion from 10^−5^ to 10^−7^ drives a ten-fold evolutionary increase in allocation to enzyme production, from 0.02 to 0.2. The evolutionary response of exoenzyme production to varying diffusion feeds back to the ecological state of the whole lattice and alters ecosystem-level function: the decomposition rate averaged across the lattice rises more than four-fold as exoenzyme production adapts to reduced diffusion (Fig. 5b), driving an 80% drop in the soil C stock per microsite averaged across the lattice (Fig. 5c). Note that the patterns in Fig. 5b and c closely match the response of the exoenzyme allocation ESS to varying diffusion (Fig. 5a), and that the error bars reflecting differences in the average values among simulations are very small compared to the differences caused by the change in *φ*. This shows that the process of evolutionary microbial adaptation can drive much larger variation in the lattice-scale ecosystem properties (decomposition rate, soil C stock) than demographic and environmental stochasticity at the microsite scale.

## Discussion

Soil microbial decomposition involves the production of exoenzymes and uptake of the products of enzyme-driven depolymerization of dead organic matter. These products form a diffusive public good, which is vulnerable to exploitation by cheaters. To elucidate conditions under which decomposition, as an outcome of microbial cooperation, is evolutionarily stable against mutations of small effects, we constructed a spatial model of soil microbe-enzyme decomposition which accounts for the finite size of microbial populations at the microscopic scale of microbial interactions.

Deterministic models of microbe-enzyme-driven decomposition were first introduced by Schimel and Weintraub^15^ for “well-mixed” systems. Here we develop a rigorous mathematical framework to show that Schimel and Weintraub’s^15^ model and subsequent variants (reviewed in ref. ^28^) are consistent with microscopic processes acting at the level of individual entities (cells, molecules). Starting from a five-compartment model including SOC and DOC molecules, cells, enzyme molecules, and enzyme–SOC molecular complexes, we found that the population size of cells and molecules and some of the stochastic process rates could be rescaled to yield^15^ four-compartment deterministic cdmz model. Note that we could not find further or alternate rescaling to reduce the dimension of the system to three compartments (cdm or cmz or dmz). Furthermore, for even simpler two-compartment models, one can prove that the equilibrium with positive cell population size is always unstable, which means that the population of cells either goes extinct or grows unboundedly (results not shown). Thus, the four-compartment structure seems to be the simplest that is consistent with the individual-level processes under consideration.

The deterministic cdmz model, however, cannot be used to capture the dynamics of a spatially explicit system in which a finite number of cells and molecules interact within their local neighborhood. From the stochastic CDMZ model we obtained the stochastic-deterministic PDMP model for local populations and interactions by assuming that the size of the molecular populations (*C*, *D*, *Z*) is typically much larger than the size of the population of cells (*M*). A spatially explicit model can then be assembled by coupling hybrid models to form a lattice of microsites. Microsite and lattice-level parameters can be specified to capture the millimeter and centimeter scale, respectively, which distinguishes this model from previous individual-based simulation models of decomposition^17–20^. In particular, the model can accommodate changes in the strength of competition within a population of individuals of the same phenotype by modifying the size of microsites, and between colonies of different strains by modifying the size of the lattice. By modeling the dynamics of the cell population and decomposition within and between microsites, we can take an evolutionary stance and address the effect of spatially heterogeneous population size and growth on the dynamics of invasion of a mutant genotype in the established population of the wild-type (resident) strain.

It has long been known that environmental spatial structure can promote cooperation by facilitating benefit-sharing among cooperators. This was shown originally for pairwise interactions and later in the case of diffusive public goods. However, early models of diffusive public goods^4,43,44^ represented space only implicitly and were therefore limited in their ability to identify conditions for the evolutionary stability of cooperation. Using a spatially explicit individual-based model of enzymatic litter decomposition^17^, backed up the expectation that the rate of products diffusion was key to the stability of cooperation. This and subsequent related models^18–20,23,45^ however, focused on competition between two or a small set of exoenzyme production genotypes, e.g. a producing strain and a non-producing (“pure cheater”) strain. Our analysis goes further by predicting the evolutionary dynamics of exoenzyme production as a quantitative trait, which varies continuously due to random mutation of small effect.

Just like soil diffusion was identified as a critical factor for the stability of a producing strain against invasion by non-producers^17,23^, our model shows that the diffusion rate determines the evolutionarily stable investment in exoenzyme production. The evolutionary stability of exoenzyme production against mutation of small effects requires the segregation of genotypes at the finest spatial scale, here the scale of microsites. In our model such segregation occurs as a consequence of linking dispersal to micro-disturbances that “open” microsites to colonization. Using a continuous representation of space, Dobay et al.^23^ also concluded that the limited diffusion of public goods favors the stability of cooperative strains against “pure cheaters”. Their continuous-space model assumes that dispersing cells remain at the periphery of existing colonies, thus in effect preventing mixing of different strains at the smallest model scale, as in our model. Whether low diffusion might also promote coexistence of strains differing in exoenzyme production rate warrants further investigation. Coexistence was not observed in our simulations, but it might occur in regions of the parameter space that we did not explore. Otherwise, instances of coexistence of producing and non-producing strains reported by Allison^17^ and Kaiser et al.^19,20^ would likely be evolutionarily unstable and/or inaccessible to evolution by mutation of small effects.

Finally, our model shows how variation in evolutionarily stable exoenzyme production feeds back to ecosystem macroscopic properties such as the decomposition rate and SOC stock at lattice scale. The model predicts that if environmental change, such as variation in soil physical properties or precipitation, drives changes in soil diffusion, then the microbial community may respond evolutionarily, and in return, the microorganisms’ evolutionary, adaptive response may substantially impact ecosystem function. Previous models investigated how soil functional properties such as decomposition, heterotrophic respiration, and carbon stock, respond to variation in soil moisture due to variable precipitation^46,47^. Focusing on experimental data from semi-arid savannah-type ecosystem subject to contrasted precipitation regimes, Zhang et al.^47^ used model-data assimilation to demonstrate the importance of water saturation as a control of enzyme activity and DOC uptake, and of the accumulation and storage of enzymes and DOC (that is temporarily inaccessible to microbes) in the dry soil pores during dry periods. Our results show that microbial evolution of exoenzyme production, in and of itself, can drive strong ecosystem responses to variation in soil diffusion, due e.g. to variation in soil moisture. Droughts that affect soil diffusion may also elicit microbial physiological responses^48^ such as higher investment in osmolyte production, possibly at the expense of exoenzyme production^49^; extensions of our model could evaluate the consequences for soil carbon loss. Additionally, one could explore the relative effect on decomposition and heterotrophic respiration of microbial physiological^46,47^ and evolutionary responses to the spatial heterogeneity of soil water distribution. Elaborating on Melbourne and Chesson’s^50^ theory of scale transition, recent work by Chakrawal et al.^51^ establishes a powerful framework to incorporate soil heterogeneity in models of decomposition. Their mathematical techniques could be used for the further up-scaling of our model, from the lattice (centimetric) scale to larger soil patches, taking into account the abiotic heterogeneity that exists at such larger scales.

We conclude that large ecosystem effects may result from the evolutionary adaptive response of microbial populations to changes in soil abiotic properties like diffusion. This calls for a more general investigation of the large-scale ecosystem consequences of soil microbial evolution in response to global environmental change, such as climate warming. The thermal dependence of microbe-enzyme biochemical processes involved in decomposition can radically change the global projections of soil C in response to climate warming^52^. Future research is warranted to evaluate how microbial evolutionary adaptation to warming may further alter global projections of terrestrial carbon cycling.

## Methods

### Construction of the five-compartment model

Here we explain the construction of the five-compartment model (Fig. 1a). This is step 1 among the four steps described in “Results” section (subsection “Ecosystem dynamics at microsite scale”). We use upper bars in our initial notations to indicate parameters prior to rescaling.

The five-compartment model captures the stochastic processes acting at the level of *C*, *D*, *M*, *Z*, *X* entities (molecules, cells) (Fig. 1a) within a microsite. Dynamics of *C*, *D*, *M*, *Z*, *X* occur in continuous time. *M*_*t*_ is the number of cells at time *t*. *C*_*t*_, *D*_*t*_, *Z*_*t*_ are the numbers of SOC molecules, DOC molecules, and enzyme molecules respectively. *X*_*t*_ is the number of complexes formed by an enzyme molecule binding a SOC molecule. There are constant external sources of SOC and DOC. When a cell dies, a fraction *p* of the molecules released are recycled into SOC, while the rest is recycled into DOC. A fraction *l* of dead microbes and deactivated enzymes may be lost due to leaching.

We denote by *α* the structural cost of a cell, which is the equivalent in number of DOC molecules of one cell (without storage). We denote by $\alpha ^{\prime}$ the energetic cost of a cell, which is the number of DOC molecules consumed to produce the energy needed for the synthesis reactions involved in the production of one cell. We denote by *β* the equivalent in number of DOC molecules of one SOC molecule, and the structural and energetic cost of producing one molecule of enzyme by *ρ* and $\rho ^{\prime}$, respectively. We assume that the energetic costs are carbon released by cells as CO_2_ (cell respiration) that diffuses out of the system instantly. We define the biomass production fraction and enzyme allocation fraction as

1 $${\bar{\gamma }}_{M}:=\frac{1}{\alpha +\alpha ^{\prime} },\quad {\bar{\gamma }}_{Z}:=\frac{1}{\rho +\rho ^{\prime} }.$$

The event times are given by independent exponential random variables whose parameters are defined by event rates (Supplementary Tables 2–4). These event rates give an approximation of the average frequency of each event. The rates of cell growth and enzyme production depend on the trait *φ*. Once a cell has doubled its initial size, reproduction occurs by releasing the mother cell at its initial size, and the daughter cell at its same size. The cell must therefore take up and store its structural and energetic cost, $(\alpha +\alpha ^{\prime} )$, in DOC molecules in order to reproduce. We denote *N* the number of uptake events before reproduction. The number of DOC molecules taken up at each uptake event is then $(\alpha +\alpha ^{\prime} )/N$, hence the notation ${{\bf{1}}}_{\{D\ge (\alpha +\alpha ^{\prime} )/N\}}$ which equals 1 if $D\ge (\alpha +\alpha ^{\prime} )/N$, and 0 otherwise. The same notation, ${{\bf{1}}}_{\{D\ge \rho +\rho ^{\prime} \}}$, is used for the production event of an enzyme molecule. Uptake is stochastic, but reproduction is deterministic, which means that when a cell has performed *N* uptake events, it reproduces with probability 1. A larger *N* means a larger number of uptake events between 2 reproduction events, which also means less DOC molecules taken up at each uptake event. The model tracks the dynamics of the number of cells, SOC, DOC, enzyme molecules, and also of the DOC stored in each cell.

Enzyme–substrate complexes form at rate ${\bar{\lambda }}^{k}$ as one enzyme molecule (e.g. cellulase) bind one SOC molecule (e.g. cellulose). A complex may either dissociate (with no decomposition) at rate ${\bar{\lambda }}_{-1}^{\varepsilon }$, or react at rate ${\bar{\mu }}^{\varepsilon }$ and convert the molecule of SOC into *β* molecule of DOC while the enzyme is released and free again to react with new molecules of SOC (Supplementary Table 3).

System size *k* does not appear in this system of equations, yet it enters the volume-dependent parameters of the model, *I*_*C*_, *I*_*D*_, *θ*, and *K*_*m*_. We denote *V* the unit volume of soil that contains on average one microbial cell and the corresponding equilibrium of carbon mass of SOC, DOC and enzymes. The system size *k* is the number of well-mixed unit volumes in one microsite, which determines the number of cells sharing SOC, DOC, and enzymes. The volume of one microsite is therefore *k* × *V*. Increasing *k* amounts to increasing microsite volume, the number of cells sharing resources in one microsite, the amount of resources per microsite, and the volume-dependent parameters, such as the amount of SOC entering a microsite per unit of time, *I*_*C*_. In our analysis, the unit volume *V* is fixed, and we vary *k* to investigate the effect of microsite volume on the system’s eco-evolutionary dynamics. With very large *k*, the hybrid model can be approximated by a fully deterministic model which takes the form of a system of four ordinary differential equations (see Supplementary Information 3.3 and Supplementary Fig. 1), similar to the microbial decomposition model first introduced by Allison et al.^53^. However, empirical data suggest that *k* is of the order of 10–100^54^. When *k* = 1, there is only one cell in the microsite, which volume is *V* defined as the unit soil volume expected to contain a single cell. A value of *k* greater than 1 means that each microsite contains *k* cells and *k* times the amount of SOC, DOC and enzyme molecules of 1 cell; thus, the microsite volume is *k* × *V*, and volume-dependent parameters are rescaled by *k*. Specifically, there are four volume-dependent parameters: the external input of *C*, ${\bar{I}}_{C}^{k}$, the external input of *D*, ${\bar{I}}_{D}^{k}$, the half-saturation constant of DOC uptake, ${\bar{K}}_{m}^{k}$, and the encounter intensity of two given SOC and enzyme molecules, ${\bar{\lambda }}^{k}$. The external inputs increase proportionally with the volume of the microsite, while the encounter intensity of two given molecules in a microsite decreases as its volume increases. The half-saturation is inversely proportional to the affinity between a given cell *M* and a given DOC molecule *d*, which decreases with increasing microsite volume. We thus obtain the following scaling relationships:

2 $${\bar{I}}_{C}^{k}=k{\bar{I}}_{C},\ {\bar{I}}_{D}^{k}=k{\bar{I}}_{D},\ {\bar{\lambda }}^{k}=\frac{\lambda }{k}\ {\rm{and}}\ {\bar{K}}_{m}^{k}=k{\bar{K}}_{m}.$$

In our simulations, we generally assume that *k* is equal to 10, to match the empirical observation that (cells) in soil habitat tend to interact with 10 to 100 other cells at all time^54^.

### Derivation of the hybrid model

In Supplementary Information 3, we present the next two steps (2 and 3 in “Ecosystem dynamics at microsite scale” of “Results” section) to derive the hybrid model on which all our results are based. In Supplementary Information 3.1, we explain how the dynamics generated by the five-compartment model can be captured with a reduced model with four-state variables (step 2). In Supplementary Information 3.2, we explain how the stochastic-deterministic PDMP model can be derived from the stochastic four-state variable model (step 3). In the hybrid model (step 4), only cell death remains stochastic, and cell dynamics is measured in unit of number of individuals (*M*), while other entities are now in carbon mass unit. The rescaled SOC, DOC and enzyme abundances are denoted with lower case letters *c*, *d*, and *z*.

### Simulation algorithm for the hybrid spatial model

One technical benefit of the hybrid model is its much greater computational tractability. Here we describe the algorithm used to perform simulations of the hybrid model. We ran the model on single microsite to produce the simulations reported in Fig. 2. We ran the model on a 10 × 10 lattice of microsites for the subsequent figures. The algorithm is based on the Gillepsie algorithm^55^ as used in Champagnat et al.^37^, Fournier and Méléard^56^, which straightforwardly extends to the simulation of PDMPs.

To couple PDMP models across microsites, we account for the DOC and dispersal of cells between adjacent microsites. The DOC diffusion between microsites is modeled by approximating a continuous diffusion with a Euler scheme in which time is discretized with a fixed time step interval, *τ*_diff_. *τ*_diff_ is chosen sufficiently small to provide a fine enough discretization of the DOC diffusion.

A simulation starts with a given amount of *M*, *z*, *c*, and *d* in each microsite at time *t* = 0, while the initial amount of DOC stored within each cell is determined uniformly at random. Two stochastic events (death of a cell) may not occur at the same time. Assume that the process has been computed until time *t*_*i*_; to continue the computation to time *t*_*i*+1_, we proceed as follows.

First, we simulate *T*, an exponential random variable with parameter *r*(*t*_*i*_) = *d*_*M*_*M*(*t*_*i*_), which corresponds to the death rate of the total cell population at time *t*_*i*_ (*M*(*t*_*i*_) being the total number of cells on the entire lattice). We then compute

$${t}_{i+1}:={t}_{i}+\min \left(T,{\tau }_{{\rm{diff}}}\right).$$

To obtain *c*(*t*_*i*+1_), *d*(*t*_*i*+1_), and *z*(*t*_*i*+1_) in each microsite at time *t*_*i*+1_, and the variation in amount of DOC stored within a cell in the corresponding microsite, we use a Euler scheme that solves the dynamical system

$$\left\{\begin{array}{l}\hskip -136pt \dot{c}(t)={I}_{C}-{l}_{C}c-\theta zc,\\ \dot{d}(t)={I}_{D}-{l}_{D}d+\theta zc+(1-l){d}_{Z}z-{V}_{\max }\frac{d}{{K}_{m}{\,}+{\,}d}{\omega }_{M}M,\\ \hskip -84pt \dot{z}(t)=\varphi {\gamma }_{Z}{V}_{\max }\frac{d}{{K}_{m}{\,}+{\,}d}{\omega }_{M}M-{d}_{Z}z,\\ \hskip -93pt \dot{\Delta }(t)=(1-\varphi ){\gamma }_{M}{V}_{\max }\frac{d}{{K}_{m}{\,}+{\,}d}{\omega }_{M},\end{array}\right.$$

in each microsite between *t*_*i*_ and *t*_*i*+1_, where *M* is the number of cells in the microsite at time *t*_*i*_, Δ gives the amount variation of DOC stored within a cell, Δ(*t*_*i*_) = 0 and the other initial conditions are the biomass of *c*, *d*, *z* in the corresponding microsite at time *t*_*i*_.

Note that, within a microsite, the variation of stored DOC is the same for all cells and corresponds to Δ(*t*_*i*+1_). Hence, this amount is added to the amount of DOC stored within each cell living in the corresponding microsite. If, for a cell *j*, the resulting amount *S*_*j*_(*T*_*i*_) + Δ(*t*_*i*+1_) is over *ω*_*M*_, a new cell appears. The amount of stored DOC within the new cell and the mother cell is then updated: *S*_*j*_(*t*_*i*+1_) = (*S*_*j*_(*T*_*i*_) + Δ(*t*_*i*+1_) − *ω*_*M*_)/2. Otherwise, *S*_*j*_(*t*_*i*+1_) = *S*_*j*_(*T*_*i*_) + Δ(*t*_*i*+1_). To determine the position of the new cell, the following steps are taken:

- A uniform random variable *ϑ*_1_ in [0, 1] is simulated.
- If *ϑ*_1_ < 1 − *p*_*disp*_, the new cell is added to the mother cell microsite.
- Otherwise, the new cell disperses:
  - If empty microsites are available in the four nearest microsites, the new cell is added to one of them, drawn randomly.
  - Otherwise, a uniform random variable *ϑ*_2_ in [0, 1] is simulated.
    - If *ϑ*_2_ < 1 − *p*_*open*_, the new cell is added to the mother cell microsite.
    - If *ϑ*_2_ ≥ 1 − *p*_*open*_, a micro-disturbance happens. That is, one of the four nearest microsites is chosen at random and all cells in the microsite die. These cells are removed from the population, an amount of (1 − *l*)(1 − *p*)*ω*_*M*_*M* is added to variable *d* and an amount of (1 − *l*)*pω*_*M*_*M* is added to variable *c* in this microsite (where *M* is the number of cells that died in the event). Finally, the new cell is placed in this microsite.

If several birth events happen during the same time interval [*t*_*i*_, *t*_*i*+1_], the new cells are relocated one after another in a randomly and uniformly drawn order.

At this point, no diffusion of DOC has yet taken place. The next step in the algorithm therefore consists in calculating the diffusion of DOC as driven by a step of the Euler scheme associated with the diffusion equation

$$\frac{{\rm{d}}}{{\rm{d}}t}d(x,t)={\sigma }_{{\rm{diff}}}\Delta d(x,t).$$

This is done by updating the computed DOC biomass *d*_*j*,*l*_(*t*_*i*+1_) in the microsite of the *j*th column and the *l*th line and of the lattice by replacing it with

$${d}_{j,l}({t}_{i+1})+\frac{{\sigma }_{{\rm{diff}}}\cdot \tau }{{(Vk)}^{2/3}} \Big({d}_{j+1,l}({t}_{i+1})+{d}_{j-1,l}({t}_{i+1})+{d}_{j,l+1}({t}_{i+1})\\ \,\,\, +\,{d}_{j,l-1}({t}_{i+1})-4* {d}_{j,l}({t}_{i+1})\Big).$$

Finally we test for a cell dying at time *t*_*i*+1_:

- If *t*_*i*+1_ − *t*_*i*_ = *T*, then a cell dies at time *t*_*i*+1_. It is chosen uniformly at random among all alive cells and it is removed from the population. At the same time, an amount of (1 − *l*)*pω*_*M*_ is added to variable *d* and an amount of (1 − *l*)(1 − *p*)*ω*_*M*_ is added to variable *c* in the corresponding microsite.
- If *t*_*i*+1_ − *t*_*i*_ = *τ*_diff_ (i.e. *T* > *τ*_diff_), no cell dies.

All steps are then repeated until a set time is reached or all cells died.

### Parameter values

We used default parameter values from previous modeling literature. Most existing models of microbial decomposition are deterministic^28,29^; therefore, to compare them and their parameters tothe hybrid model, we established a fully deterministic version of our model, and then reversed the successive renormalizations to obtain the hybrid model’s parameters as functions of the parameters of the fully deterministic model. The derivation of the fully deterministic model, called cdmz, from the stochastic CDMZ model is presented in Supplementary Information 3.2 and takes the form of a system of differential equations, see equations (S.5). Mapping these equations to similar models in Allison^53^, German et al.^57^, Hagerty et al.^58^, Schimel and Weintraub^15^ provided us with default parameter values listed in Table 1. In addition, the structural and energetic costs (*α*s and *ρ*s) are calculated from the masses (*ω*s) and production fractions (*γ*s) of the variables (see Supplementary Equation (S.3)).

The decomposition rate *θ* is calculated as $\frac{{V}_{\mathop{\max }_{{\rm{decomposition}}}}}{{K}_{{m}_{{\rm{decomposition}}}}}$ from Allison et al.’s^53^ model. We ignore the input of DOC to focus on the internal mechanism of DOC production driven by microbial enzyme decomposition. Additionally, a sensitivity analysis (see below, and Supplementary Table 1) of the deterministic approximation cdmz model (Supplementary Information 3.3) shows that *I*_*D*_ has very little influence on the state variables, including the SOC stock, *c*. SOC and DOC are assumed to leach out of the system at the same rate. Dead microbes and deactivated enzymes are recycled half into SOC and the other half into DOC (*p* = 0.5). The values of *p*_*mut*_ and *σ*_*mut*_ reflect the assumption that mutations are rare and small^59^.

For the change of unit from biomass to individual entities (*ω*s), we used *Bacillus subtilis* or *B. clausii*, cellulase, cellulose and glucose as our baseline. We estimated the mass of 1 DOC molecule with the mass of 1 molecule of glucose. We estimated the mass of 1 SOC molecule from the approximation that 1 molecule of cellulose contains about 10^3^ molecules of glucose. We estimated the mass of 1 enzyme molecule by assuming that 1 molecule of cellulase contains about as much carbon as 1 molecule of cellulose. Finally, we estimated the mass of 1 cell based on the results from biomass estimations of soil samples (with various methods, such as CFI, CFE, SIR) that there are about 4 × 10^8^ active cells in 1 cm^3^ of bulk soil, which weigh 0.1 mg in carbon^60^.

### Eco-evolutionary analysis

We perform extensive simulations of the resulting eco-evolutionary dynamics (joint dynamics of the ecological variables and trait distribution) at the microscale of single sites.

At the lattice scale, we analyze the evolution of exoenzyme production by using the spatial version of the hybrid model. To circumvent the issue of prohibitive computation time, we predict the long-term stationary state of eco-evolutionary dynamics by using an adaptive dynamics approach^26,38^. To this end, rather than simulating the cell population with a continuous flow of new mutants, we parallelize the simulations of an ensemble of pairwise contests between only two slightly different strains at a time, one strain taken as resident (initially at stationary state) and the other as the initially rare, mutant strain. The direction and strength of selection on the evolving trait is then predicted by invasion fitness (intrinsic population growth rate from low density) of the mutant strain competing against the resident strain^26,27^. Based on the mathematical theory of adaptive dynamics in finite populations^42^, a proxy for invasion fitness is given by the product of the mutant probability of survival with the long-term population growth rate of surviving mutant populations. The rationale is that deleterious mutants may experience positive growth due to genetic drift, but their probability of survival is low; in contrast, advantageous mutants that differ only slightly from the resident strain tend to grow slowly, but their survival probability is high. An evolutionarily stable strategy (ESS) can then be estimated from pairwise contest simulations, as a trait value that no nearby mutant can invade, i.e. for which the estimated invasion fitness proxy of nearby mutants is close to zero. When invasion fitness indicates that for any given resident strain, mutants closer to the ESS outcompete the resident, then the ESS is attractive and the system’s eco-evolutionary dynamics converge to the ESS. In this case, the (attractive) ESS predicts the outcome of the full eco-evolutionary dynamics under the assumption of mutations of small effects^37^.

### Simulation of resident-mutant interaction in the spatial model

We chose a standard lattice size of 10 × 10 microsites (in their related but non-evolutionary models, Allison^17^ and Kaiser et al.^19,20^ used lattices of 100 × 100 microsites) to avoid the prohibitive computation time of slow population spread across larger lattices. The observed patterns of spread conform to a relatively smooth invasion front, which makes us confident that the invasion capacity of a mutant phenotype is adequately captured by the dynamics following its introduction in a 10 × 10 lattice. The probability of invasion of a mutant might decrease with increasing lattice size if a larger lattice tended to increase the risk of stochastic extinction by drift; however, (1) extinction by drift is actually more likely to occur in the initial phase of invasion, when the spread is still limited to a small part of the lattice, and (2) even if this were the case, the consequence may be a flatter fitness landscape around the ESS, but the direction of selection towards the ESS should not be affected.

We start each simulation with a monomorphic population (all cells have the same *φ* value). All four variables *c*, *d*, *M*, *z* are initialized at the steady state values predicted by the deterministic model (Supplementary Eq. (S.5)) with the corresponding values of *k* and *φ*. Mutants are initially located at the center of the lattice (changing the initial location does not modify the final fraction of mutants in the lattice). On the edges of the lattice we impose Neumann boundary conditions whereby there is no disappearance of dispersing cells or DOC diffusing out of the lattice. Diffusion is then a transfer of *c*, *d*, *z* between the three (instead of four) neighboring microsites (or two for corner microsites) and the center microsite. Likewise, dispersing cells may move into one of three microsites (or two for corner microsites) instead of four. Looking for edge effects, we carefully monitor the potential accumulation of substrates or individuals at the boundary or in corners. No substantial such effect manifested over the time span over which mutant invasion develops.

To optimize simulation time, we assume that mutants occur initially at 5% frequency in the introduction microsite. We run simulations for (resident, mutant) pairs with ±0.05 difference in trait value *φ*. From the final frequency of mutants we compute the mutant exponential growth rate, and average over 20 simulation replicates. All simulations are different due to demographic stochasticity. The total simulation time of all parallelized runs was 10^7^ h (about 1000 years).

### Reporting summary

Further information on research design is available in the Nature Research Reporting Summary linked to this article.

## Supplementary information

Supplementary Information

Reporting Summary

## Data Availability

The simulations and figures that support the findings of this study were coded with C++ and R. The code is available at https://zenodo.org/badge/latestdoi/217151545^61^.
