# Supplementary material for: A multi-scale eco-evolutionary model of cooperation reveals how microbial adaptation influences soil decomposition
Source: Commun Biol. 2020 Sep 21;3:520. doi: 10.1038/s42003-020-01198-4 (PMC7505970; doi:10.1038/s42003-020-01198-4)
Supplement: Supplementary file 1 — Supplementary Information [file 42003_2020_1198_MOESM1_ESM.pdf]

Supplementary Information

A multi-scale eco-evolutionary model of cooperation reveals how microbial adaptation influences soil decomposition

Contents

1 Supplementary Figures

1.1 Supplementary Figure 1. Effect of system size k on the dynamics of the cell population (M) in a single microsite, in the absence of mutation.

1.2 Supplementary Figure 2. Effect of exoenzyme production φ on the dynamics of total cell population M and total mass of z, c, and d across a multi-site lattice, in the absence of mutation.

1.3 Supplementary Figure 3. Effect of lattice size on the pattern of selection on exoenzyme production at different soil diffusion rates.

2 Supplementary Tables

2.1 Supplementary Table 1. Sensitivity analysis.

2.2 Supplementary Table 2. Events involving the enzyme-SOC complexes (X) characteristic of the 5-compartment model.

2.3 Supplementary Table 3. Events involving C, D, M, Z in both the 5- and 4-compartment model.

2.4 Supplementary Table 4. Poisson Point Processes encoding all the events involved in the CDMZX and CDMZ models.

3 Supplementary Notes

3.1 Reduction of the stochastic model from five (CDMZX model) to four state variables (CDMZ model)

3.2 Derivation of the stochastic-deterministic model, (PDMP), by rescaling the stochastic CDMZ model

3.3 Deterministic approximation of the stochastic CDMZ model

3.4 Sensitivity analysis

3.5 Numerical comparison of the hybrid and deterministic (cdmz) models

3.6 Rigorous proof of Theorem 1

1 Supplementary Figures

1.1 Supplementary Figure 1. Effect of system size k on the dynamics of the cell population (M) in a single microsite, in the absence of mutation.

(a) k=10

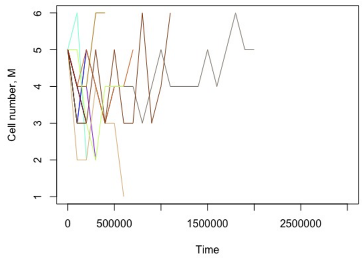

(b) k=12

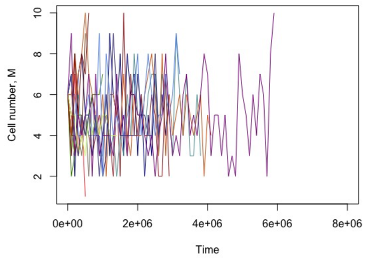

(c) k=15

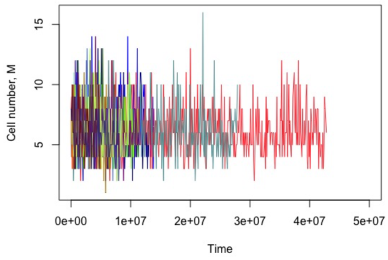

(d) k=20

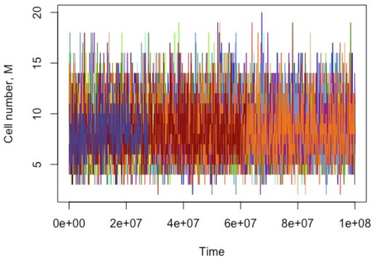

(e) k=1000

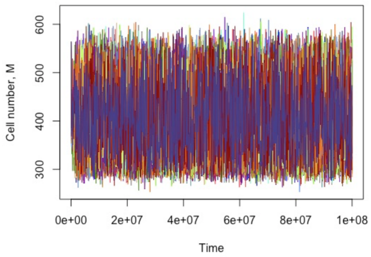

Supplementary Figure 1. Effect of system size k on the dynamics of the cell population (M) in a single microsite and in the absence of mutation. The model used here is the hybrid model for a single microsite. Five values of k are used between 10 and 1,000. For each value of k, twenty simulation runs are reported; each run is colored differently. Simulations stop when the cell population reaches zero. Parameter values: All constant parameters are set to their default values (Table 1), initial conditions are adjusted to $\varphi = 0.5$, and $T_{\max} = 10^8$.

1.2 Supplementary Figure 2. Effect of exoenzyme production φ on the dynamics of total cell population M and total mass of z, c, and d across a multi-site lattice, in the absence of mutation.

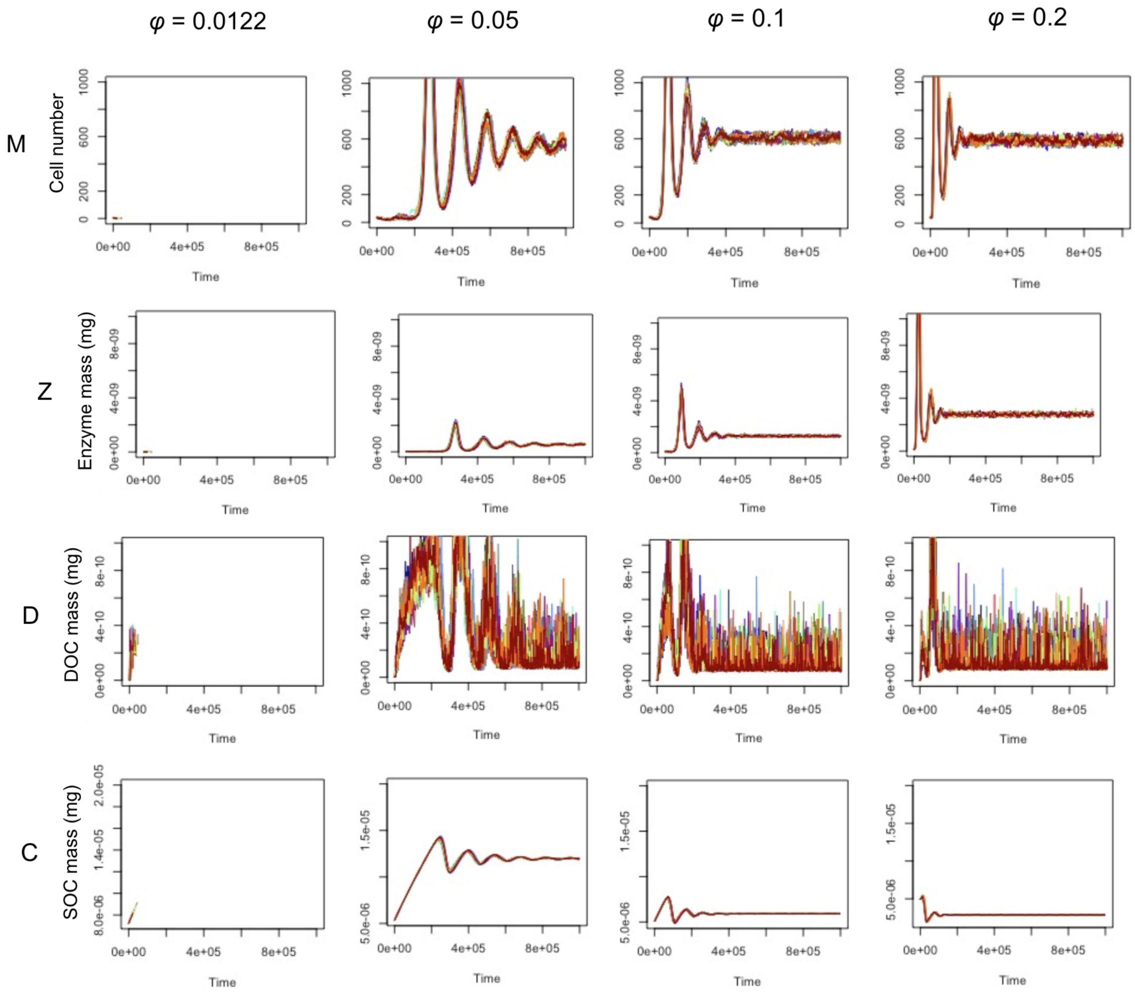

Supplementary Figure 2. Effect of exoenzyme production φ on the dynamics of total cell population M and total mass of z, c, and d across a multisite lattice, in the absence of mutation. The model used is a 100-microsites (10 x 10 lattice) hybrid model. Parameter values: $k = 10$ for all microsites, and all constant parameters are set to the default values (Supplementary Table 1). Initially, only 5 central microsites are occupied by cells; the population then colonizes the lattice, and lattice-scale c, d, M, z converge to equilibrium values. for each φ value. In the 5 central microsites, c, d, M, z are set to the equilibrium predicted by the deterministic approximation model set with the corresponding φ value. In the other 95 microsites, $d = M = z = 0$ and $c = 5 \times 10^{-5} mg$, which corresponds to the φ-independent trivial equilibrium (presented in Supplementary Information 3.5).

1.3 Supplementary Figure 3. Effect of lattice size on the pattern of selection on exoenzyme production at different soil diffusion rates.

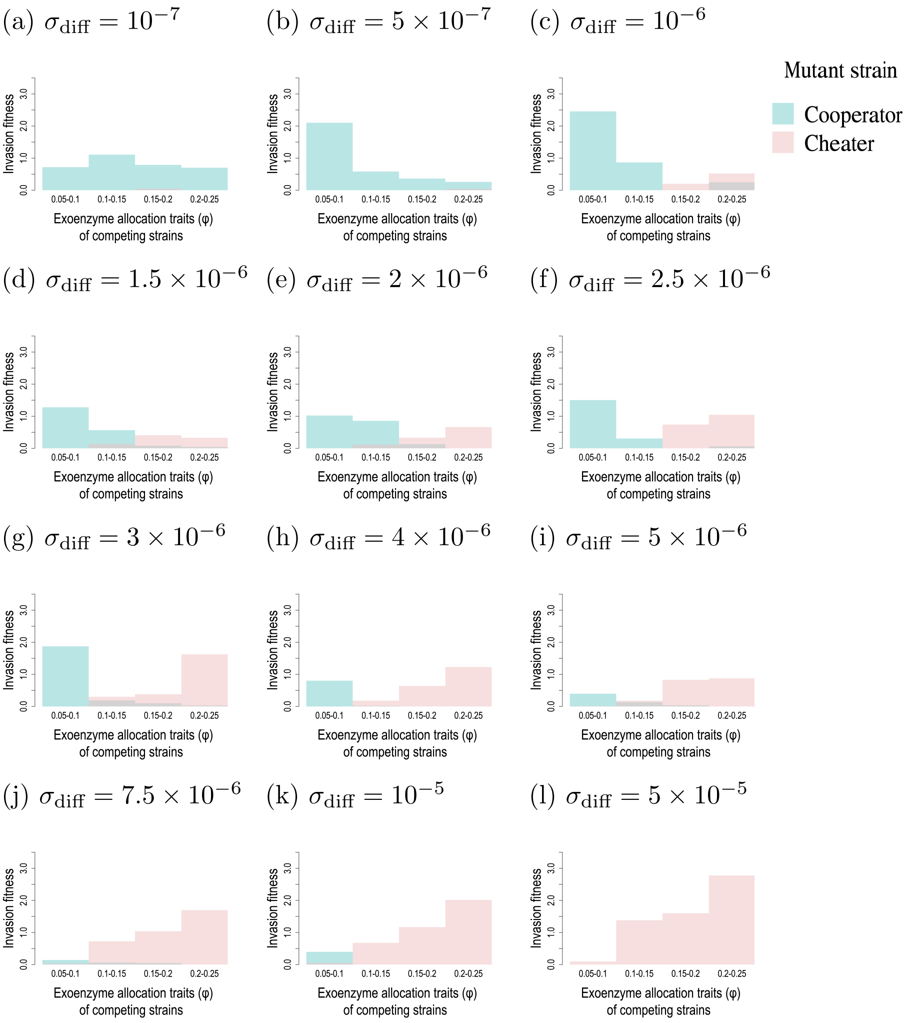

Supplementary Figure 3. Patterns of selection on exoenzyme production at different soil diffusion rates on a larger, 6 x 6 lattice of microsites, than in Fig. 4. Each graph shows the mutant invasion fitness across pairwise resident-mutant competing strains. Red bars show invasion fitness of the cheater strain taken as mutant (with the lower φ value in the competing pair); blue bars show invasion fitness of the cooperator strain taken as mutant (with the higher φ value in the competing pair). Positive invasion fitness of cheater mutants (red bars) indicate selection against exoenzyme production. Positive invasion fitness of cooperator mutants (blue bars) indicate selection in favor of exoenzyme production. For each diffusion rate, the ESS is bracketed between the maximum φ value for which the cooperator mutant has positive invasion fitness, and the minimum φ value for which the cheater mutant has positive invasion fitness. Parameters and number of simulations are the same as for Fig. 4. See the caption of Fig. 4 for more details.

2 Supplementary Tables

2.1 Supplementary Table 1. Sensitivity analysis.

			Sensitivity (absolute value)			
Parameter	Low value	High value	c_{eq}	d_{eq}	m_{eq}	z_{eq}
φ	0.19	0.95	1.13	1.00	1.00	0.99
γ_M	0.19	0.95	0.23	1.00	1.55	0.55
γ_Z	0.19	0.95	1.04	0	0.18	1.18
d_M	2×10^{-5}	2×10^{-3}	0	1.00	1.00	0
d_Z	2×10^{-4}	2×10^{-3}	1.00	0	0.19	0.19
V_{max}	0.042	4.2	0	1.00	0	0
θ	0.03	3	1.00	0	0.19	0.19
K_m	0.19	0.95	0	1.00	0	0
I_C	0.19	0.95	0	0	1.19	1.19
I_D	0.19	0.95	0.17	0	0.39	0.39
l_C	0.19	0.95	0	0	0.19	0.19
l_D	0.19	0.95	0	0	0	0
l	0.19	0.95	0.12	0	0.17	0.17
p	0.19	0.95	0.07	0	0.22	0.22

Supplementary Table 1. Unidimensional sensitivity analysis of the deterministic cdmz model to model parameters.

2.2 Supplementary Table 2. Events involving the enzyme-SOC complexes (X) characteristic of the 5-compartment model.

Event	Event rate
Formation of 1 complex X from 1 Z and 1 C:<br>$(C, D, M, Z, X) \mapsto (C-1, D, M, Z-1, X+1)$	$\bar{\lambda}^k ZC$
Dissociation of 1 complex X into 1 Z and 1 C:<br>$(C, D, M, Z, X) \mapsto (C+1, D, M, Z+1, X-1)$	$\bar{\lambda}^{\varepsilon}_{-1} X$
Depolymerization of 1 C into β D (decomposition) from the effect of Z on C in complex X<br>$(C, D, M, Z, X) \mapsto (C, D+\beta, M, Z+1, X-1)$	$\bar{\mu}^{\varepsilon} X$

Supplementary Table 2. Events involving enzyme-SOC complexes (X) in the CDMZX model. Individual-level events and event rates.

2.3 Supplementary Table 3. Events involving C, D, M, Z in both the 5- and 4-compartment model.

Event	Event rate
Events relative to M	
M grows and accumulates an equivalent of α/N molecules of DOC, gives birth to an offspring if its stock of carbon is equal to α $D \mapsto D - \frac{\alpha+\alpha'}{N}$	$N(1-\varphi)\bar{\gamma}_M \bar{V}_{max} \frac{D}{K_m^k+D} \mathbf{1}_{\left\{D \geq \frac{\alpha+\alpha'}{N}\right\}}$
M dies $C \mapsto C + \lfloor (1-l)p\frac{\alpha}{\beta} \rfloor$, $D \mapsto D + \lfloor (1-l)(1-p)\alpha \rfloor$	$\bar{d}_M$
M produces 1 Z $D \mapsto D - (\rho+\rho')$	$\varphi \bar{\gamma}_Z \bar{V}_{max} \frac{D}{K_m^k+D} \mathbf{1}_{\{D \geq \rho+\rho'\}}$
Events specific to Z, C, D	
Deactivation of 1 Z: $(C, D, M, Z) \mapsto (C, D + \lfloor (1-l)\rho \rfloor, M, Z-1)$	$\bar{d}_Z Z$
External input of 1 C: $(C, D, M, Z) \mapsto (C+1, D, M, Z)$	$\bar{I}_C^k$
Loss of 1 C due to leaching: $(C, D, M, Z) \mapsto (C-1, D, M, Z)$	$\bar{l}_C C$
External input of 1 D: $(C, D, M, Z) \mapsto (C, D+1, M, Z)$	$\bar{I}_D^k$
Loss of 1 D due to leaching: $(C, D, M, Z) \mapsto (C, D-1, M, Z)$	$\bar{l}_D D$
Event specific to CDMZ model	
Depolymerization of 1 C into β D (decomposition) through enzymatic reaction $(C, D, M, Z) \mapsto (C-1, D+\beta, M, Z+1)$	$\bar{\theta}^k Z C$

Supplementary Table 3. Events and event rates in both stochastic models (CDMZ and CDMZX). Cells are characterized by their trait value φ.

2.4 Supplementary Table 4. Poisson Point Processes encoding all the events involved in the CDMZX and CDMZ models.

Event type number	Description	Rate
1	appearance of a C	$r_1^\varepsilon(t) = \bar{I}_C$
2	disappearance of a C	$r_2^\varepsilon(t) = \bar{l}_C C^\varepsilon$
3	appearance of a D	$r_3^\varepsilon(t) = \bar{I}_D$
4	disappearance of a D	$r_4^\varepsilon(t) = \bar{l}_D D^\varepsilon$
5	birth of a M	$r_5^\varepsilon(t) = (1-\varphi)\bar{\gamma}_M \bar{V}_{max} \frac{D^\varepsilon}{\bar{K}_m + D^\varepsilon} M^\varepsilon \mathbf{1}_{\{D^\varepsilon \geq \alpha+\alpha'\}}$
6	death of a M	$r_6^\varepsilon(t) = \bar{d}_M M^\varepsilon$
7	production of a Z	$r_7^\varepsilon(t) = \varphi\bar{\gamma}_Z \bar{V}_{max} \frac{D^\varepsilon}{\bar{K}_m + D^\varepsilon} M^\varepsilon \mathbf{1}_{\{D^\varepsilon \geq \rho+\rho'\}}$
8	deactivation of a Z	$r_8^\varepsilon(t) = \bar{d}_Z Z^\varepsilon$
9	formation of a X	$r_9^\varepsilon(t) = \bar{\lambda} Z^\varepsilon C^\varepsilon$
10	dissociation of a X	$r_{10}^\varepsilon(t) = \bar{\lambda}_{-1}^\varepsilon X^\varepsilon$
11	decomposition of a X	$r_{11}^\varepsilon(t) = \bar{\mu}^\varepsilon X^\varepsilon$

Supplementary Table 4. Poisson Point Processes that encodes the 11 different types of events of the model. Event type numbers, biological descriptions and event rates.

3 Supplementary Notes

In Supplementary Information §3, we derive the hybrid model that we simulated to obtain the results presented in the main text. In the Methods, we presented the construction of the five-compartment model (see Figure 1a). Here we explain the next two steps (steps 2 and 3) listed in the Results section (subsection Ecosystem dyanmics at microsite scale) of the main text. Specifically, in Supplementary Information 3.1, we explain how the dynamics generated by the five-compartment model can be captured with a reduced model with four state variables. In Supplementary Information 3.2, we explain how the stochastic-deterministic model (PDMP model) can be derived from the stochastic four-state variable model. We use upper bars in our initial notations to indicate parameters prior to rescaling.

3.1 Reduction of the stochastic model from five (CDMZX model) to four state variables (CDMZ model)

We introduce $\varepsilon > 0$ such that

$$\bar{\lambda}_{-1}^{\varepsilon} = \frac{1}{\varepsilon}\bar{\lambda}_{-1} \quad \text{and} \quad \bar{\mu}^{\varepsilon} = \frac{1}{\varepsilon}\bar{\mu}. \tag{S.1}$$

where $\bar{\lambda}_{-1}$ is the dissociation rate of X into C and Z (unsuccessful decomposition), and $\bar{\mu}$ the depolymerization rate of X into D and Z (successful decomposition).

Let k be fixed and let

$$C^{\varepsilon}(t), D^{\varepsilon}(t), M^{\varepsilon}(t), Z^{\varepsilon}(t), X^{\varepsilon}(t)$$

designate the (stochastic) number of SOC molecules, DOC molecules, cells, enzyme molecules, and complexes at time t in the CDMZX model associated to parameters $\bar{\lambda}_{-1}^{\varepsilon}$ and $\bar{\mu}^{\varepsilon}$. Next theorem ensures that under the assumption that complex dissociation and the decomposition reaction of the complex are much faster than complex formation:

$$\bar{\lambda}_{-1}^{\varepsilon},\ \bar{\mu}^{\varepsilon} >> \bar{\lambda}^{k},$$

the stochastic CDMZX model can be simplified into the four-compartment stochastic CDMZ model with structure shown in Fig. 1b and event rates listed in Supplementary Table 3 with

$$\bar{\theta}^{k} = \bar{\lambda}^{k}\frac{\bar{\mu}}{\bar{\mu} + \bar{\lambda}_{-1}} := \frac{\bar{\theta}}{k}, \tag{S.2}$$

where $\bar{\theta}^{k}$ measures the microscopic encounter intensity of one specific SOC molecule and one specific enzyme, multiplied by the probability that a thus-formed complex X depolymerizes rather than dissociates (Supplementary Table 2).

Let

$$\mathbb{Y}^{\varepsilon} := (C^{\varepsilon} + X^{\varepsilon}, D^{\varepsilon}, M^{\varepsilon}, Z^{\varepsilon} + X^{\varepsilon}),$$

then the following result, which is proved in Supplementary Information 3.6, holds.

Theorem 1. *Assume that* (S.1) *holds and that* $(C^\varepsilon, D^\varepsilon, M^\varepsilon, Z^\varepsilon, X^\varepsilon)(0)$ *converges in* L^2 *to the deterministic vector* $(C_0, D_0, M_0, Z_0, X_0)$*, when* ε *goes to* 0*, then for any* $T \geq 0$*, the sequence of processes* $(\mathbb{Y}^\varepsilon(t), t \in [0,T])_{\varepsilon>0}$ *converges in law, in* $\mathbb{D}([0,T], \mathbb{N}^4)$ *endowed with the Skorohod topology, to* $(C, D, M, Z)_{t\in[0,T]}$ *defined by the 4-compartment model whose rates are described in Supplementary Table 3, initial condition is* (C_0, D_0, M_0, Z_0) *and* $\bar{\theta}^k$ *is defined by* (S.2)*.*

3.2 Derivation of the stochastic-deterministic model, (PDMP), by rescaling the stochastic CDMZ model

As explained in the main text Results, the biomass represented by a cell is much larger than the carbon mass of a molecule of SOC, DOC or enzyme, whereas the number of cells is much smaller than the number of SOC, DOC and enzyme molecules. Based on this, we now show that the stochastic CDMZ individual-based model can be further rescaled into a deterministic-stochastic model, where only cell dynamics remain stochastic and measured in unit of number of individuals, while other entities are now in carbon mass unit. One technical benefit of the PDMP model is its much greater computational tractability. Note that the rescaled SOC, DOC and enzyme abundances are denoted with lowercase letters c, d, and z.

Let us introduce parameter κ to rescale the carbon mass ratio, α, of one cell M to one DOC molecule D. By letting κ go to infinity, we can represent cells that are many times larger than other molecules and in contact with a very large number of molecules. To do so, we consider the process $(C^\kappa, D^\kappa, M^\kappa, Z^\kappa)$, which is similar to the four-compartments model of Supplementary Information 3.1, but with the following parameters depending on κ: $\alpha_\kappa := \kappa\alpha$, $\alpha'_\kappa := \kappa\alpha'$, $\bar{I}_C^\kappa := \kappa\bar{I}_C$, $\bar{I}_D^\kappa := \kappa\bar{I}_D$, $\bar{\theta}^\kappa := \frac{\bar{\theta}}{\kappa}$, and $\bar{K}_m^\kappa := \kappa\bar{K}_m$. Now, we are interested in the sequence

$$\left(\omega_C\frac{C_t^\kappa}{\kappa}, \omega_D\frac{D_t^\kappa}{\kappa}, M_t^\kappa, \omega_Z\frac{Z_t^\kappa}{\kappa}, t \geq 0\right)_{\kappa\geq0},$$

when κ tends to ∞. ω_C is the mean carbon mass content of one SOC molecule, and likewise for DOC (ω_D), cells (ω_M), and enzyme (ω_Z). The ω parameters are related to α, β and ρ according to:

$$\beta = \frac{\omega_C}{\omega_D}, \quad \alpha = \frac{\omega_M}{\omega_D}, \quad \text{and} \quad \rho = \frac{\omega_Z}{\omega_D}. \tag{S.3}$$

Using the ω parameters allows the system of equations to be expressed in biomass and not in molecular density, which would be less meaningful and more difficult to interpret.

With further rescaling (such that all parameters of the system are expressed in biomass) and notations:

$$\begin{array}{llllllllllll} I_C & := & \omega_C\bar{I}_C, & I_D & := & \omega_D\bar{I}_D, & K_m & := & \omega_D\bar{K}_m, & & & \\ \theta & := & \frac{1}{\omega_Z}\bar{\theta}, & V_{max} & := & \frac{\omega_D}{\omega_M}\bar{V}_{max}, & \gamma_M & := & \frac{\omega_M}{\omega_D}\bar{\gamma}_M, & \gamma_Z & := & \frac{\omega_Z}{\omega_D}\bar{\gamma}_Z, \\ d_M & := & \bar{d}_M, & d_Z & := & \bar{d}_Z, & l_C & := & \bar{l}_C, & l_D & := & \bar{l}_D, \end{array} \tag{S.4}$$

a direct application of Theorem 3.1 of Crudu et al. [2012] gives the following theorem.

Theorem 2. *Assume that* $\left(\omega_C \frac{C^\kappa(0)}{\kappa}, \omega_D \frac{D^\kappa(0)}{\kappa}, M^\kappa(0), \omega_Z \frac{Z^\kappa(0)}{\kappa}\right)$ *converges to a deterministic vector* (c_0, d_0, M_0, z_0)*, then the sequence of processes*

$$\left(\omega_C \frac{C_t^\kappa}{\kappa}, \omega_D \frac{D_t^\kappa}{\kappa}, M_t^\kappa, \omega_Z \frac{Z_t^\kappa}{\kappa}, t \geq 0\right)$$

converges in distribution (in the Skorohod topology), when κ *goes to* $+\infty$*, to the distribution of a PDMP characterized by its infinitesimal generator* $\mathcal{A}$*, which describes a time-homogeneous Markov process (whose dynamics depend on time intervals but not on time itself). It is defined as follow: for any continuous and bounded function* f *in* $\mathbb{R}^4$*,*

$$\begin{aligned}
&\mathcal{A}f(c,d,M,z) = \\
&\quad (I_C - l_C c - \theta z c)\,\frac{\partial f(M,z,c,d)}{\partial c} \\
&\quad + \left(I_D - l_D d + \theta z c + (1-\varepsilon) d_Z z - \varphi \omega_M V_{max} \frac{d}{K_m + d} M\right) \frac{\partial f(M,z,c,d)}{\partial d} \\
&\quad + (1-\varphi)\gamma_M V_{max} \frac{d}{K_m + d} M \mathbf{1}_{\{d \geq \omega_D(\alpha+\alpha')\}} \Big[f\left(M+1, z, c, d - \omega_D(\alpha+\alpha')\right) - f(M,z,c,d) \Big] \\
&\quad + d_M M \Big[f\left(M-1, z, c + (1-\varepsilon) p \alpha \omega_D, d + (1-\varepsilon)(1-p)\alpha\omega_D\right) - f(M,z,c,d) \Big] \\
&\quad + \left(\varphi \omega_M \gamma_Z V_{max} \frac{d}{K_m + d} M - d_Z z\right) \frac{\partial f(M,z,c,d)}{\partial z}.
\end{aligned}$$

This generator corresponds precisely to the generator of the PDMP described in the third item of the Results section (subsection **Ecosystem dynamics at microsite scale**) of the main text.

For the sake of simplicity, the theorem has been written in the case where $N = 1$ in the CDMZ model, however, it can be directly adapted for $N > 1$. In our context, approximating the microscopic model by the limiting PDMP is justified due to the large values of $\alpha = 10^{10}$ and $\alpha' = 2.33 \cdot 10^{10}$ compared to β, ρ and ρ' (less than 10^4). Finally, when N is really large, we consider that the growth of all cells is also deterministic (step 4 in **Ecosystem dynamics at microsite scale** of the Results section). The rationale is explained in the Results section; a rigorous proof of this approximation goes beyond the scope of this paper.

3.3 Deterministic approximation of the stochastic CDMZ model

Microbial models in the literature are deterministic. To compare them and their parameters to the hybrid model, we establish a fully deterministic version of our model, and then reverse the successive renormalizations to obtain the hybrid model's parameters as functions of the parameters of the fully deterministic model. For the sake of keeping the mathematical derivation as simple as possible, we present the derivation of the deterministic model starting from the full stochastic CDMZ model; similar derivations can be unfolded from the PDMP or the hybrid model. The fully deterministic model takes the form of a system of ordinary differential equations, similar to Schimel and Weintraub [2003] seminal model of litter decomposition (see also Abs and Ferrière [2020], Wieder et al. [2015]).

Here we assume that all cells have the same trait value, φ, so that there is only one type of cells in the system, and we take a large system size, k. If (0.2) holds, we prove that the stochastic CDMZ model can be approximated by the following cdmz deterministic model

$$\begin{cases} \dfrac{dc}{dt} = I_C - l_C c + (1-l)pd_M m - \theta zc \\ \dfrac{dd}{dt} = I_D - l_D d + \theta zc + (1-l)\left[(1-p)d_M m + d_Z z\right] - V_{max}\dfrac{d}{K_m + d}m, \\ \dfrac{dm}{dt} = (1-\varphi)\gamma_M V_{max}\dfrac{d}{K_m+d}m - d_M m \\ \dfrac{dz}{dt} = \varphi\gamma_Z V_{max}\dfrac{d}{K_m+d}m - d_Z z \end{cases} \tag{S.5}$$

where c, d, m, and z are in carbon mass unit, and all parameters correspond to rescaled parameters defined in (S.4).

Specifically, let us denote by $(C^k(t), D^k(t), M^k(t), Z^k(t))$ the number of SOC molecules, DOC molecules, cells and enzymes molecules given by the stochastic CDMZ model presented in Supplementary Information 3.1 with parameters depending on k. The following lemma results from a direct application of Chapter 11 in Ethier and Kurtz [2009].

Lemma 3.1. *Assume that* (0.2) *holds and that*

$$\left(\omega_C\frac{C^k(0)}{k}, \omega_D\frac{D^k(0)}{k}, \omega_M\frac{M^k(0)}{k}, \omega_Z\frac{Z^k(0)}{k}\right) \underset{k\to+\infty}{\longrightarrow} (c(0), d(0), m(0), z(0)) \in [0,+\infty)^4,$$

then for any $T \geq 0$,

$$\lim_{k\to+\infty}\sup_{t\leq T}\left\|\left(\omega_C\frac{C^k(t)}{k}, \omega_D\frac{D^k(t)}{k}, \omega_M\frac{M^k(t)}{k}, \omega_Z\frac{Z^k(t)}{k}\right) - (c(t), d(t), m(t), z(t))\right\|_\infty = 0,$$

where the limit stands in probability, $\|.\|_\infty$ *denotes the* L^∞*-norm on* $\mathbb{R}^4$ *and* (c, d, m, z) *is the unique solution to* (S.5) *with initial condition* $(c(0), d(0), m(0), z(0))$.

3.4 Sensitivity analysis

We performed a unidimensional sensitivity analysis of c, d, m, z to the model parameters using the equilibria of the deterministic approximation presented in Supplementary Information 3.3. We used the measure of sensitivity from Allison et al. [2010], and a ten-fold magnitude range for all parameters, except for the fraction parameters tested between 0.1 and 0.9. Sensitivities below 10^{-3} are rounded to 0. Interestingly, the SOC stock is mainly sensitive to the parameters involved in the microbial allocation strategy, the enzyme allocation fraction φ, enzyme production efficiency, γ_Z, and enzyme deactivation rate, d_Z, and to the encounter and decomposition rates, θ.

To assess the robustness of the ESS dependence on diffusion to lattice size (Fig. 5a), while circumventing the computation time issue, we performed simulations with a *smaller* lattice (6 x 6 microsites). In these simulations, we introduced mutants in two microsites to maintain the initial mutant frequency at 5%, and we used the same system size ($k = 10$) to maintain the same local

competitive environment. Supplementary Figure 3 shows that invasion fitness values are very similar to those obtained with the 10 x 10 lattice, which confirms that variation in lattice size may have little effect on the model predictions.

3.5 Numerical comparison of the hybrid and deterministic (cdmz) models

The deterministic model corresponds to a large k version of the hybrid model for a single microsite. Its ecological dynamics defined in (S.5) can be solved analytically. There are one or three equilibria depending on the value of φ. At the "trivial" equilibrium, there are no active microbes or enzymes ($m_{eq1} = z_{eq1} = 0$), SOC and DOC are fixed by the balance of external inputs and leaching ($c_{eq1} = I_C/l_C$ and $d_{eq1} = I_D/l_D$). This equilibrium is always locally stable. The other two equilibria exist when c, d, m, z at both equilibria are all positive, which depends on φ belonging to a certain interval ($\varphi_{min,existence} \leq \varphi \leq \varphi_{max,existence}$). One of the two positive equilibria is always unstable, and the other equilibrium is stable over a smaller interval $\varphi_{min,stability} \leq \varphi \leq \varphi_{max,stability}$, and a limit cycle over $\varphi_{max,stability} \leq \varphi \leq \varphi_{max,existence}$, and bifurcating into a limit cycle on $\varphi_{min,existence} \leq \varphi \leq \varphi_{min,stability}$. For the default parameters values (Supplementary Table 1), $\varphi_{min,existence} = 0.01211$, $\varphi_{min,stability} = 0.01212$, $\varphi_{max,stability} = 0.9969$, and $\varphi_{max,stability} = 0.9984$. Thus, $\varphi_{min,existence}$ and $\varphi_{min,stability}$ on the one hand, and $\varphi_{max,existence}$ and $\varphi_{max,stability}$ on the other, virtually coincide; and the latter is approximately 1, making all cases of long-term oscillatory dynamics extremely marginal and of little biological relevance. However, the existence of oscillatory attractors can leave a "footprint" on short-term, transient dynamics, and this was observed indeed (Supplementary Figure 2).

For viable values of φ (between 0.01212 and 0.9969 given our default parameter values), the microbial population in the single-microsite hybrid model does not go extinct during the simulated time provided the system size k is large enough, in spite of strong oscillations around the stationary state. However, at smaller k, the average microbial biomass m decreases, and stochasticity combined with fluctuations can lead to rapid extinction, resulting in a smaller range of viable φ (Supplementary Figure 1). There is a threshold on k under which the range of viable φ departs from deterministic predictions. This threshold decreases in the multi-microsite, lattice version of the model (Supplementary Figure 2), as a manifestation of a metapopulation rescue effect: because local extinctions do not occur simultaneously, the metapopulation (at lattice scale) can survive local microsite population extinctions, with the added effect that dead cells from an extinct site are recycled into resource, which feeds and promote survival of neighbouring populations.

3.6 Rigorous proof of Theorem 1

In Supplementary Information 3.6, we provide a rigorous proof of Theorem 1. To reduce the CDMZX model, the main difficulty arises because we do not have the classical Skorohod convergence in distribution for the process $(C^\varepsilon, D^\varepsilon, M^\varepsilon, Z^\varepsilon, X^\varepsilon, t \in [0,T])_{\varepsilon>0}$. For reader convenience, we drop the notation k in the whole proof. Indeed, when ε is small, there will be some

really close jumps of X^ε when a complex is formed and almost immediately dissociated or decomposed. It is why we are interested in the process

$$\mathbb{Y}^\varepsilon := (C^\varepsilon + X^\varepsilon, D^\varepsilon, M^\varepsilon, Z^\varepsilon + X^\varepsilon),$$

and we prove the convergence of the sequence of processes $(\mathbb{Y}^\varepsilon(t), t \in [0,T])_{\varepsilon>0}$ in law, in $\mathbb{D}([0,T], \mathbb{N}^4)$ endowed with the Skorohod topology, to $(C, D, M, Z)_{t\in[0,T]}$ defined by the 4-compartment model of Supplementary Information 3.1.

Here we take $N = 1$, but the proof could be generalized $N > 1$ by introducing multiple cell stages describing the state of stored cell resource.

Proof. **Step 1**: The first step is to prove the tightness of sequence $(\mathbb{Y}^\varepsilon)_{\varepsilon>0}$ in $\mathbb{D}([0,T], \mathbb{N}^4)$. To this aim, we denote the jumps set of process $(\mathbb{Y}^\varepsilon(t), t \in [0,T])$ by

$$\{J_j^\varepsilon\}_{j\geq 1} = \{t \in [0,T], \mathbb{Y}^\varepsilon(t-) \neq \mathbb{Y}^\varepsilon(t)\}. \tag{S.6}$$

Note that $\mathbb{Y}^\varepsilon$ is càdlàg (right continous with left limits), hence the definition (S.6). As any jump of $\mathbb{Y}^\varepsilon$ is of size 1, the tightness of $\mathbb{Y}^\varepsilon$ follows from the two conditions:

i) $\lim_{a\to+\infty} \limsup_{\varepsilon\to 0} \mathbb{P}(\|\mathbb{Y}^\varepsilon\|_\infty \geq a) = 0$,

ii) $\lim_{\delta\to 0} \limsup_{\varepsilon\to 0} \mathbb{P}(\exists j \geq 0, J_{j+1}^\varepsilon - J_j^\varepsilon \leq \delta) = 0$.

Indeed, these two conditions directly imply the two conditions of Theorem 13.2 in the book of Billingsley [2013], which ensures tightness.

To prove i), we introduce $N_{Tot}^\varepsilon = \beta C^\varepsilon + D^\varepsilon + \alpha M^\varepsilon + \rho Z^\varepsilon + (\rho+\beta) X^\varepsilon$ the total equivalent number of DOC molecules in the system at any time. Since the only external sources of carbon are inputs of SOC and DOC, N_{Tot}^ε is stochastically bounded from above by

$$\sup_{s\leq T} N_{Tot}^\varepsilon(s) \preceq N_{Tot}^\varepsilon(0) + \mathcal{P}((\bar{I}_D + \beta \bar{I}_C)T) =: N_{\max}^\varepsilon, \tag{S.7}$$

where $\mathcal{P}((\bar{I}_D + \beta \bar{I}_C)T)$ is a Poisson random variable with parameter $(\bar{I}_D + \beta \bar{I}_C)T$. From the assumption on the initial conditions, we deduce immediately that the random variable $N_{\max}^\varepsilon$ is L^2-integrable and that for ε sufficiently small, there exists $C_0 > 0$ such that

$$\mathbb{E}\left[N_{\max}^\varepsilon\right] + \mathbb{E}\left[(N_{\max}^\varepsilon)^2\right] \leq C_0. \tag{S.8}$$

Moreover, since α, β and ρ are greater than 1, we obtain from Markov inequality,

$$\mathbb{P}(\|\mathbb{Y}^\varepsilon\|_\infty \geq a) \leq \mathbb{P}(N_{\max}^\varepsilon \geq a) \leq \frac{1}{a} C_0,$$

for any ε sufficiently small. This ends the proof of i).

We now deal with ii). Let us set $\eta > 0$. First of all, note that we can focus the study on the set $\{X^\varepsilon(0) = 0\}$. Indeed, from the assumption on the initial condition, for any ε small enough, $\mathbb{P}(X^\varepsilon(0) \geq 1) \leq \eta$. Hence

$$\mathbb{P}(\exists j \geq 0, J_{j+1}^\varepsilon - J_j^\varepsilon \leq \delta) \ \leq \ \eta + \mathbb{P}_0(\exists j \geq 0, J_{j+1}^\varepsilon - J_j^\varepsilon \leq \delta), \tag{S.9}$$

where for any set $\mathcal{A}$, $\mathbb{P}_0(\mathcal{A}) = \mathbb{P}(\mathcal{A}|X^\varepsilon(0) = 0)$. In what follows, we restrict our focus on $\{X^\varepsilon(0) = 0\}$.

Then, we count the number of jumps of $\mathbb{Y}^\varepsilon$. Note that any jump of $\mathbb{Y}^\varepsilon$ is also a jump of $(C^\varepsilon, D^\varepsilon, M^\varepsilon, Z^\varepsilon, X^\varepsilon)$. Thus, we count the jumps number of the latter. As originally done by Fournier and Méléard [2004], it is convenient to represent a trajectory of individual-based processes as the unique solution of a system of stochastic differential equations driven by Poisson point measures. To this aim, we introduce a collection of 11 independent Poisson Point Processes $(N^i(\mathrm{d}s, \mathrm{d}\theta))_{i=1,..,11}$ on $[0,\infty)^2$ with intensity $\mathrm{d}s\mathrm{d}\theta$ and independent of ε, which will be used to encode the 11 different types of events of the process $(C^\varepsilon, D^\varepsilon, M^\varepsilon, Z^\varepsilon, X^\varepsilon)$. We also denote all rates of this process by $(r_i^\varepsilon(t), t \in [0,T])_{i=1,..,11}$ and indexed in Supplementary Table 4.
Note that only the events of type 1 to 8 and 11 correspond to jumps of $\mathbb{Y}^\varepsilon$. Hence, the jumps number of $\mathbb{Y}^\varepsilon$ can be bounded stochastically by

$$\sharp\{J_j^\varepsilon\} \preceq \sum_{i\in\{1,..,8,11\}} \int_0^T \int_{\mathbb{R}^+} \mathbf{1}_{\{\theta \le r_i^\varepsilon(s-)\}} N^i(\mathrm{d}s, \mathrm{d}\theta). \tag{S.10}$$

The only problem comes from the last rate $\bar{\mu}^\varepsilon X^\varepsilon$, which is unbounded when ε goes to 0. However $\bar{\mu}^\varepsilon X^\varepsilon = 0$ as soon as there is no complex X in the system, and complexes are created with the encounter of a Z and a C (9-th rate). Thus, we immediately conclude that

$$\int_0^T \int_{\mathbb{R}^+} \mathbf{1}_{\{\theta \le \bar{\mu}^\varepsilon X^\varepsilon(s-)\}} N^{11}(\mathrm{d}s, \mathrm{d}\theta) \le \int_0^T \int_{\mathbb{R}^+} \mathbf{1}_{\{\theta \le \bar{\lambda} Z^\varepsilon(s-) C^\varepsilon(s-)\}} N^9(\mathrm{d}s, \mathrm{d}\theta).$$

In addition with (S.10), (S.7) and (S.8), we deduce, if ε is small enough that

$$\begin{aligned}
\mathbb{P}_0(\sharp\{J_j^\varepsilon\} > n) &\le \sum_{i=1}^{9} \mathbb{P}_0\left(\int_0^T \int_{\mathbb{R}^+} \mathbf{1}_{\{\theta \le r_i^\varepsilon(s-)\}} N^i(\mathrm{d}s, \mathrm{d}\theta) \ge \frac{n}{9}\right) \\
&\le \frac{9}{n} T \sum_{i=1}^{9} \mathbb{E}_0\left[\sup_{s\in[0,T]} r_i^\varepsilon(s)\right] \\
&\le \frac{9T}{n}\left(\bar{I}_C + \bar{I}_D + C_1 \mathbb{E}_0\left[N_{\max}^\varepsilon\right] + \bar{\lambda}\mathbb{E}_0\left[(N_{\max}^\varepsilon)^2\right]\right) \\
&\le \frac{9T}{n} C_2 \underset{n\to+\infty}{\longrightarrow} 0,
\end{aligned} \tag{S.11}$$

with $C_1 := \bar{\gamma}_M \bar{V}_{max} + \bar{d}_M + \bar{\gamma}_Z \bar{V}_{max} + \bar{d}_Z + \bar{l}_C + \bar{l}_D$ and $C_2 := \bar{I}_C + \bar{I}_D + C_1 C_0 + \bar{\lambda} C_0$. We fix $n := \lfloor 9TC_2/\eta \rfloor + 1$ such that the last r.h.s. is smaller than η. Thus,

$$\begin{aligned}
\mathbb{P}_0(\exists j \ge 0, J_{j+1}^\varepsilon - J_j^\varepsilon \le \delta) \ &\le \ \mathbb{P}_0(\sharp\{J_j^\varepsilon\} > n) + \mathbb{P}_0\left(\exists j \in \{1,..,n-1\}\ J_{j+1}^\varepsilon - J_j^\varepsilon \le \delta,\ \ \sharp\{J_j^\varepsilon\} \le n\right) \\
&\le \ \eta + \sum_{j=1}^{n-1} \mathbb{P}_0(J_{j+1}^\varepsilon - J_j^\varepsilon \le \delta)
\end{aligned} \tag{S.12}$$

Moreover, for any $j \in \{1,..,n-1\}$,

$$\mathbb{P}_0(J_{j+1}^\varepsilon - J_j^\varepsilon \le \delta) \ \le \ \mathbb{P}_0(J_{j+1}^\varepsilon - J_j^\varepsilon \le \delta | X^\varepsilon(J_j^\varepsilon) = 0) + \mathbb{P}_0(X^\varepsilon(J_j^\varepsilon) \ge 1) \tag{S.13}$$

The first term of the r.h.s of (S.13) can be bounding using the Markov property of $(C^\varepsilon, D^\varepsilon, M^\varepsilon, Z^\varepsilon, X^\varepsilon)$. Indeed, the two last types of events (10 and 11) can not occur after time J_j^ε and before any other jumps, since $X^\varepsilon(J_j^\varepsilon) = 0$. Hence

$$\begin{aligned}\mathbb{P}_0(J_{j+1}^\varepsilon - J_j^\varepsilon \leq \delta | X^\varepsilon(J_j^\varepsilon) = 0) &\leq \mathbb{P}_0\left(\exists i \in \{1,..,9\}, \int_0^\delta \int_{\mathbb{R}+} \mathbf{1}_{\{\theta \leq r_i^\varepsilon(s-)\}} N^i(\mathrm{d}s, \mathrm{d}\theta) \geq 1\right)\\ &\leq \delta \sum_{i=1}^9 \mathbb{E}_0\left[\sup_{s\in[0,T]} r_i^\varepsilon(s)\right] \leq \delta C_2 \leq \frac{\eta}{n},\end{aligned}$$

as soon as $\delta \leq \eta/(nC_2)$. Hence, with (S.12) and (S.13),

$$\mathbb{P}_0(\exists j \geq 0, J_{j+1}^\varepsilon - J_j^\varepsilon \leq \delta) \ \leq \ 2\eta + \sum_{j=1}^{n-1} \mathbb{P}_0(X^\varepsilon(J_j^\varepsilon) \geq 1). \tag{S.14}$$

To bound the second term of the r.h.s of (S.14), recall that the positive jumps of X^ε are not jumps of $\mathbb{Y}^\varepsilon$ and note that $X^\varepsilon(J_j^\varepsilon)$ may be greater than 1 only if there exists a positive jump of X^ε whose next event is of type 1 to 9 (and not of type 10 or 11). We denote the set of positive jumps of X^ε by

$$\{S_\ell^\varepsilon\}_{\ell\geq 1} = \{t \in [0,T], X^\varepsilon(t) - X^\varepsilon(t-) = 1\}.$$

The second term of the r.h.s of (S.14) can thus be bounded by

$$\sum_{j=1}^{n-1} \mathbb{P}_0(X^\varepsilon(J_j^\varepsilon) \geq 1) \leq \mathbb{P}_0\left(\exists \ell \geq 1, \min_{1\leq i\leq 9} \tau_i^\varepsilon(S_\ell^\varepsilon) \leq \min\{\tau_{10}^\varepsilon(S_\ell^\varepsilon), \tau_{11}^\varepsilon(S_\ell^\varepsilon)\}\right), \tag{S.15}$$

where for any $i = 1,..,10$, $\tau_i^\varepsilon(S_\ell^\varepsilon)$ is the first time event of type i after S_ℓ^ε, that is

$$\tau_i^\varepsilon(S_\ell^\varepsilon) := \inf\left\{t \geq S_\ell^\varepsilon, \int_{S_\ell^\varepsilon}^t \int_{\mathbb{R}+} \mathbf{1}_{\{\theta \leq r_i^\varepsilon(s-)\}} N^i(\mathrm{d}s, \mathrm{d}\theta) \geq 1\right\}.$$

After time S_ℓ^ε and before any other event, X^ε is obviously greater than 1. The rates r_{10}^ε and r_{11}^ε can thus be bounded from below by $\bar{\lambda}_{-1}^\varepsilon$ and $\bar{\mu}^\varepsilon$ respectively, other rates can be bounded from above using the r.v. $N_{\max}^\varepsilon$. Thus, using again (S.11), together with (S.15) and the Markov property satisfied by $(C^\varepsilon, D^\varepsilon, M^\varepsilon, Z^\varepsilon, X^\varepsilon)$, we obtain

$$\begin{aligned}\sum_{j=1}^{n-1} \mathbb{P}_0(X^\varepsilon(J_j^\varepsilon) \geq 1) &\leq \sum_{\ell=1}^{n} \mathbb{P}_0\left(\min_{1\leq i\leq 9} \tau_i^\varepsilon(S_\ell^\varepsilon) \leq \min\{\tau_{10}^\varepsilon(S_\ell^\varepsilon), \tau_{11}^\varepsilon(S_\ell^\varepsilon)\}\right) + \mathbb{P}_0(\sharp\{S_\ell^\varepsilon\} > n)\\ &\leq n\mathbb{P}_0\left(\tau \leq \mathcal{E}_{\bar{\lambda}_{-1}^\varepsilon + \bar{\mu}^\varepsilon}\right) + \eta,\end{aligned}$$

where $\mathcal{E}_{\bar{\lambda}_{-1}^\varepsilon + \bar{\mu}^\varepsilon}$ is an exponential r.v. with parameter $\bar{\lambda}_{-1}^\varepsilon + \bar{\mu}^\varepsilon$, and,

$$\tau = \inf\left\{t \geq 0, \int_0^t \int_{\mathbb{R}+} \mathbf{1}_{\{\theta \leq \bar{I}_C + \bar{I}_D + C_1 N_{\max}^\varepsilon + \bar{\lambda}(N_{\max}^\varepsilon)^2\}} N^1(\mathrm{d}s, \mathrm{d}\theta) \geq 1\right\}.$$

Hence

$$\sum_{j=1}^{n-1} \mathbb{P}_0(X^\varepsilon(J_j^\varepsilon) \geq 1) \leq n \int_0^\infty \mathbb{P}_0(\tau \leq s)\,(\bar{\lambda}_{-1}^\varepsilon + \bar{\mu}^\varepsilon) e^{-(\bar{\lambda}_{-1}^\varepsilon + \bar{\mu}^\varepsilon)s} \mathrm{d}s + \eta$$
$$\leq n\varepsilon \frac{C_2}{\bar{\lambda}_{-1} + \bar{\mu}} + \eta. \tag{S.16}$$

Finally, with (S.9), (S.14) and (S.16), we obtain

$$\limsup_{\varepsilon \to 0} \mathbb{P}(\exists j \geq 0, J_{j+1}^\varepsilon - J_j^\varepsilon \leq \delta) \;\leq\; 4\eta,$$

as soon as $\delta \leq \eta^2/(18TC_2^2)$ (as this implies that $\delta \leq \eta/(nC_2)$). This ends the proof of ii), and the one of the tightness of process $\mathbb{Y}^\varepsilon$.

Step 2: The second step is to identify the limit. As the sequence of processes $(\mathbb{Y}^\varepsilon)_{\varepsilon>0}$ is tight, it is sufficient to prove that any accumulation point has the same law. Let us take $(C, D, M, Z) \in \mathbb{D}([0,T], \mathbb{N}^4)$ the limit (in law) of a sub-sequence of $(\mathbb{Y}^\varepsilon)_{\varepsilon>0}$, that we denote also by $(\mathbb{Y}^\varepsilon)_{\varepsilon>0}$ for the sake of readability and we will denote (C, D, M, Z) by $\mathbb{Y}$. We first prove that $\mathbb{Y}$ is a Markov process and then characterize it by describing its jump rates. Note that $\{\mathbb{Y}^\varepsilon\}_{\varepsilon>0}$ are not Markov processes, however $\{(\mathbb{Y}^\varepsilon, X^\varepsilon)\}_{\varepsilon>0}$ are Markov processes.

To prove that $\mathbb{Y}$ is a Markov process, let us set $t > 0$, a sequence of $m + m'$ times $0 \leq t_1 \leq ... \leq t_m \leq t \leq s_1 \leq .. \leq s_{m'}$ and $m + m' + 1$ vectors, $y_1, .., y_m, y_t, y'_1, .., y'_{m'} \in \mathbb{N}^4$. From Dynkin's theorem, it is sufficient to prove that

$$\mathbb{P}\Big(\mathbb{Y}(s_{m'}) = y'_{m'}, .., \mathbb{Y}(s_1) = y'_1 | \mathbb{Y}(t) = y_t, \mathbb{Y}(t_m) = y_m, .., \mathbb{Y}(t_1) = y_1\Big)$$
$$= \mathbb{P}\Big(\mathbb{Y}(s_{m'}) = y'_{m'}, .., \mathbb{Y}(s_1) = y'_1 | \mathbb{Y}(t) = y_t\Big). \tag{S.17}$$

From the convergence in law and assumptions on $X^\varepsilon(0)$, we have, for any $\varepsilon > 0$,

$$\mathbb{P}\Big(\mathbb{Y}(s_{m'}) = y'_{m'},.., \mathbb{Y}(s_1) = y'_1 | \mathbb{Y}(t) = y_t, .., \mathbb{Y}(t_1) = y_1\Big)$$
$$= \lim_{\varepsilon \to 0} \mathbb{P}_0\Big(\mathbb{Y}^\varepsilon(s_{m'}) = y'_{m'}, .., \mathbb{Y}^\varepsilon(s_1) = y'_1 | \mathbb{Y}^\varepsilon(t) = y_t, .., \mathbb{Y}^\varepsilon(t_1) = y_1\Big)$$
$$= \lim_{\varepsilon \to 0} \frac{\sum_{k \geq 0} \mathbb{P}_0\Big(\mathbb{Y}^\varepsilon(s_{m'}) = y'_{m'}, .., (\mathbb{Y}^\varepsilon, X^\varepsilon)(t) = (y_t, k), .., \mathbb{Y}^\varepsilon(t_1) = y_1\Big)}{\sum_{k \geq 0} \mathbb{P}_0\Big((\mathbb{Y}^\varepsilon, X^\varepsilon)(t) = (y_t, k), .., \mathbb{Y}^\varepsilon(t_1) = y_1\Big)}. \tag{S.18}$$

Then we prove that, for ε small enough, $X^\varepsilon(t)$ is equal to 0 with a large probability. Indeed, $(X^\varepsilon(u))_{u \leq t}$ has little chance to reach 2:

$$\mathbb{P}_0(\sup_{u \leq t} X^\varepsilon(u) \geq 2) \leq \mathbb{P}_0\left(\exists \ell \geq 1, \min_{1 \leq i \leq 9} \tau_i^\varepsilon(S_\ell^\varepsilon) \leq \min\{\tau_{10}^\varepsilon(S_\ell^\varepsilon), \tau_{11}^\varepsilon(S_\ell^\varepsilon)\}\right),$$

where all terms have been defined in (S.15), and the r.h.s term has been proved to converge to 0 when ε goes to 0. It remains to prove that $X^\varepsilon(t)$ has little chance to be equal to 1 on $\{\sup_{u \leq t} X^\varepsilon(u) \leq 1\}$

$$\mathbb{P}_0\Big(X^\varepsilon(t) = 1,\, \sup_{u \leq t} X^\varepsilon(u) \leq 1\Big) \leq \mathbb{P}_0\Big(\exists \ell \geq 1,\; S_\ell^\varepsilon \leq t < S_\ell^\varepsilon + \min\{\tau_{10}^\varepsilon(S_\ell^\varepsilon), \tau_{11}^\varepsilon(S_\ell^\varepsilon)\},\; \sup_{u \leq t} X^\varepsilon(u) \leq 1\Big).$$

As previously, note that there is not an infinite number of events S_ℓ^ε in $[0,T]$ and that $\{S_\ell^\varepsilon\}_{\ell>0}$ are directly correlated to the events of type 9. As $\min\{\tau_{10}^\varepsilon(S_\ell^\varepsilon), \tau_{11}^\varepsilon(S_\ell^\varepsilon)\}$ is an exponential random variable $\mathcal{E}_{\bar{\lambda}_{-1}^\varepsilon+\bar{\mu}^\varepsilon}$, we deduce,

$$\begin{aligned}\mathbb{P}_0(X^\varepsilon(t)=1,&\{\sup_{u\le t} X^\varepsilon(u)\le 1\})\\ &\le \sum_{\ell\ge 1}^{n}\int_0^\infty \mathbb{P}_0\Big(S_\ell^\varepsilon \in]t-h,t]\Big)(\bar{\lambda}_{-1}^\varepsilon+\bar{\mu}^\varepsilon)e^{-h(\bar{\lambda}_{-1}^\varepsilon+\bar{\mu}^\varepsilon)}dh + \mathbb{P}_0\left(\sharp\{S_j^\varepsilon\}>n\right)\\ &\le n\int_0^\infty \mathbb{P}_0\Big(\int_{t-h\vee 0}^t\int_{\mathbb{R}+}\mathbf{1}_{\{\theta\le r_9^\varepsilon(s-)\}}N^9(ds,d\theta)\ge 1\Big)(\bar{\lambda}_{-1}^\varepsilon+\bar{\mu}^\varepsilon)e^{-h(\bar{\lambda}_{-1}^\varepsilon+\bar{\mu}^\varepsilon)}dh+\eta\\ &\le n\int_0^\infty h\bar{\lambda}C_0(\bar{\lambda}_{-1}^\varepsilon+\bar{\mu}^\varepsilon)e^{-h(\bar{\lambda}_{-1}^\varepsilon+\bar{\mu}^\varepsilon)}dh+\eta\\ &\le \frac{n\bar{\lambda}C_0}{\bar{\lambda}_{-1}^\varepsilon+\bar{\mu}^\varepsilon}+\eta\le 2\eta,\end{aligned}$$

as soon as ε is sufficiently small. In other words, $\mathbb{P}_0(X^\varepsilon(t)\ge 1)$ converges to 0 with ε. (S.18) becomes

$$\begin{aligned}\mathbb{P}\Big(\mathbb{Y}(s_{m'})=y'_{m'},..,\mathbb{Y}(s_1)=y'_1|\mathbb{Y}(t)=y_t,..,\mathbb{Y}(t_1)=y_1\Big)\\ &=\lim_{\varepsilon\to 0}\frac{\mathbb{P}_0\Big(\mathbb{Y}^\varepsilon(s_{m'})=y'_{m'},..,(\mathbb{Y}^\varepsilon,X^\varepsilon)(t)=(y_t,0),..,\mathbb{Y}^\varepsilon(t_1)=y_1\Big)}{\mathbb{P}_0\Big((\mathbb{Y}^\varepsilon,X^\varepsilon)(t)=(y_t,0),..,\mathbb{Y}^\varepsilon(t_1)=y_1\Big)}\\ &=\lim_{\varepsilon\to 0}\mathbb{P}_0\Big(\mathbb{Y}^\varepsilon(s_{m'})=y'_{m'},..|(\mathbb{Y}^\varepsilon,X^\varepsilon)(t)=(y_t,0),..,\mathbb{Y}^\varepsilon(t_1)=y_1\Big)\\ &=\lim_{\varepsilon\to 0}\mathbb{P}_0\Big(\mathbb{Y}^\varepsilon(s_{m'})=y'_{m'},..|(\mathbb{Y}^\varepsilon,X^\varepsilon)(t)=(y_t,0)\Big),\end{aligned}\tag{S.19}$$

where we used the Markov property of $(\mathbb{Y}^\varepsilon, X^\varepsilon)$. Using same ideas, it is straightforward to prove that $\mathbb{P}\Big(\mathbb{Y}(s_{m'})=y'_{m'},..|\mathbb{Y}(t)=y_t\Big)$ is also equal to the last term of (S.19), hence (S.17) and the Markov property of $\mathbb{Y}$.

It remains to describe the transition rate matrix of $\mathbb{Y}$. To this aim, for any $y, y'\in\mathbb{N}^4$, we study the limits

$$\lim_{t\to 0}\mathbb{P}\Big(\mathbb{Y}(t)=y'|\mathbb{Y}(0)=y\Big).$$

From what we have seen before (notably that the events of type 1 to 8 are not really affected by the presence of the fast species X^ε), it is straightforward that, in the limiting process $\mathbb{Y}$, there exist eight types of events with rates $\bar{I}_C$ (appearance of a C molecule), $\bar{l}_C C$ (disappearance of a C molecule), $\bar{I}_D$ (appearance of a D molecule), $\bar{l}_D D$ (disappearance of a D molecule), $(1-\varphi)\bar{\gamma}_M\bar{V}_{max}\frac{D^\varepsilon}{K_m+D}M\mathbf{1}_{\{D\ge\alpha+\alpha'\}}$ (birth of a M cell), $\bar{d}_M M$ (death of a M cell), $\varphi\bar{\gamma}_Z\bar{V}_{max}\frac{D}{K_m+D}M\mathbf{1}_{\{D\ge\rho+\rho'\}}$ (production of a Z molecule), $\bar{d}_Z Z$ (deactivation of a Z molecule). We now deal with the last three types of events. However, we have seen that when a event of type 9 occurs, an event of type 10 or 11 occurs immediately after (such that the formed complex

disappears or dissociates). In the limit, both events are simultaneous and

$$\begin{aligned}
&\mathbb{P}\Big(\mathbb{Y}(t) = (c_0-1, d_0+\beta, m_0, z_0)|\mathbb{Y}(0) = (c_0, d_0, m_0, z_0)\Big) \\
&= \lim_{\varepsilon\to 0}\mathbb{P}\Big(\mathbb{Y}^\varepsilon(t) = (c_0-1, d_0+\beta, m_0, z_0)|\mathbb{Y}^\varepsilon(0) = (c_0, d_0, m_0, z_0)\Big) \\
&= \lim_{\varepsilon\to 0}\mathbb{P}\Big(\mathbb{Y}^\varepsilon(t) = (c_0-1, d_0+\beta, m_0, z_0)|\mathbb{Y}^\varepsilon(0) = (c_0, d_0, m_0, z_0)\Big)
\end{aligned}$$

It remains to characterize the jumps rate of $\mathbb{Y}$. Let us start with a birth of a M. As done previously (see (S.18)-(S.19)), we have

$$\begin{aligned}
&\mathbb{P}\Big(\mathbb{Y}(t+h) = (c, d-(\alpha+\alpha'), m+1, z)\Big|\mathbb{Y}(t) = (c,d,m,z)\Big) \\
&\qquad = \lim_{\varepsilon\to 0}\mathbb{P}_0\Big((\mathbb{Y}^\varepsilon, X^\varepsilon)(t+h) = (c,d,m+1,z,0)\Big|(\mathbb{Y}^\varepsilon, X^\varepsilon)(t) = (c,d,m,z,0)\Big).
\end{aligned}$$

Using the jumps rate of $(\mathbb{Y}^\varepsilon, X^\varepsilon)$, we deduce directly

$$\mathbb{P}\Big(\mathbb{Y}(t+h) = (c, d-(\alpha+\alpha'), m+1, z)\Big|\mathbb{Y}(t) = (c,d,m,z)\Big) = (1-\varphi)\bar{\gamma}_M\bar{V}_{max}\frac{d}{\bar{K}_m+d}m\mathbf{1}_{\{d\geq\alpha+\alpha'\}}h + o(h).$$

The same can be done with the (dis)appearance of a C, the (dis)appearance of a D, the death of a M, the production of a Z, and the deactivation of a Z, where the actions of the complexes do not intervene. And we find the rate given by Theorem (1) The only problem may come from the decomposition of a C into β D:

$$\begin{aligned}
&\mathbb{P}\Big(\mathbb{Y}(t+h) = (c-1, d+\beta, m+1, z)\Big|\mathbb{Y}(t) = (c,d,m,z)\Big) \\
&= \lim_{\varepsilon\to 0}\mathbb{P}_0\Big((\mathbb{Y}^\varepsilon, X^\varepsilon)(t+h) = (c-1, d+\beta, m, z, 0)\Big|(\mathbb{Y}^\varepsilon, X^\varepsilon)(t) = (c,d,m,z,0)\Big) \\
&= \lim_{\varepsilon\to 0}\mathbb{P}_0\Big((\mathbb{Y}^\varepsilon, X^\varepsilon)(h) = (c-1, d+\beta, m, z, 0)\Big|(\mathbb{Y}^\varepsilon, X^\varepsilon)(0) = (c,d,m,z,0)\Big) \\
&= \lim_{\varepsilon\to 0}\mathbb{P}_0\Big(S_1^\varepsilon \leq h, \tau_{11}^\varepsilon(S_1^\varepsilon) \leq \min_{1\leq i\leq 10}\tau_i^\varepsilon(S_1^\varepsilon))\Big).
\end{aligned}$$

As we proved before that $\mathbb{P}_0\left(\min_{1\leq i\leq 9}\tau_i^\varepsilon(S_1^\varepsilon) \leq \tau_{10}^\varepsilon(S_1^\varepsilon)\right)$ converges to 0 with ε (see (S.16)), we have

$$\begin{aligned}
&\mathbb{P}\Big(\mathbb{Y}(t+h) = (c-1, d+\beta, m+1, z)\Big|\mathbb{Y}(t) = (c,d,m,z)\Big) \\
&= \lim_{\varepsilon\to 0}\mathbb{P}_0\Big(S_1^\varepsilon \leq h, \tau_{11}^\varepsilon(S_1^\varepsilon) \leq \tau_{10}^\varepsilon(S_1^\varepsilon))\Big) \\
&= \lim_{\varepsilon\to 0}\left(\bar{\lambda}zc \times \frac{\bar{\mu}^\varepsilon}{\bar{\mu}^\varepsilon + \bar{\lambda}_{-1}^\varepsilon}h + o(h)\right) \\
&= \bar{\theta}zch + o(h).
\end{aligned}$$

□

References

Elsa Abs and Régis Ferrière. Modeling microbial dynamics and soil respiration, effect of climate change. in biogeochemical cycles: Ecological drivers and environmental impact. American Geophysical Union, 2020.

Steven D Allison, Matthew D Wallenstein, and Mark A Bradford. Soil-carbon response to warming dependent on microbial physiology. Nature Geoscience, 3(5):336, 2010.

Patrick Billingsley. Convergence of probability measures. John Wiley & Sons, 2013.

Alina Crudu, Arnaud Debussche, Aurélie Muller, Ovidiu Radulescu, et al. Convergence of stochastic gene networks to hybrid piecewise deterministic processes. The Annals of Applied Probability, 22(5):1822–1859, 2012.

Stewart N Ethier and Thomas G Kurtz. Markov processes: characterization and convergence, volume 282. John Wiley & Sons, 2009.

Nicolas Fournier and Sylvie Méléard. A microscopic probabilistic description of a locally regulated population and macroscopic approximations. The Annals of Applied Probability, 14(4): 1880–1919, 2004.

Joshua P Schimel and Michael N Weintraub. The implications of exoenzyme activity on microbial carbon and nitrogen limitation in soil: a theoretical model. Soil Biology and Biochemistry, 35(4): 549–563, 2003.

William R Wieder, Steven D Allison, Eric A Davidson, Katerina Georgiou, Oleksandra Hararuk, Yujie He, Francesca Hopkins, Yiqi Luo, Matthew J Smith, Benjamin Sulman, et al. Explicitly representing soil microbial processes in earth system models. Global Biogeochemical Cycles, 29 (10):1782–1800, 2015.
